# Long-term outcomes and quality of life of patients with Hirschsprung disease: a systematic review and meta-analysis

**DOI:** 10.1186/s12876-020-01208-z

**Published:** 2020-03-12

**Authors:** Ying Dai, Yongfang Deng, Yan Lin, Runxian Ouyang, Le Li

**Affiliations:** 1 Department of Pediatric Surgery, Guangzhou Women and Children’s Medical Center, Guangzhou Medical University, No. 9 Jinsui Road, Guangzhou, Guangdong Province China; 2 Department of Obstetrics and Gynecology, Guangzhou Women and Children’s Medical Center, Guangzhou Medical University, Guangzhou, China; 3 Department of Nursing, Guangzhou Women and Children’s Medical Center, Guangzhou Medical University, Guangzhou, China

**Keywords:** Children, Constipation, Follow-up, Fecal incontinence, Hirschsprung disease, Long-term outcome, Prevalence, Quality of life

## Abstract

**Background:**

Advances in surgical techniques and perioperative care have improved the short- and mid-term postoperative outcomes of patients with Hirschsprung disease (HD). However, the long-term outcomes of these patients (older than 10 years) have not been fully investigated. The aim of this systematic review is to clarify the prevalence of long-term outcomes and the quality of life of these patients.

**Methods:**

PubMed, AMED, Cochrane Library, CINAHL and PsycINFO databases were searched from inception to October 2018, following the Meta-analysis of Observational Studies in Epidemiology (MOOSE) guideline. Original studies reporting the outcomes of patients older than ten years with HD were selected and reviewed. The overall prevalence of fecal incontinence, constipation, bowel function score, bladder dysfunction symptoms, and patients’ quality of life were extracted from the included studies and pooled through the random-effects meta-analysis model. The heterogeneity and variation in the pooled estimations were evaluated by Cochrane’s Q test and the I^2^ test. The sensitivity analysis was conducted by the sequential omission of individual studies. Publication bias was evaluated by Egger’s linear regression test. The whole procedure was conducted with Stata (version 14).

**Results:**

In total, 3406 articles were identified from the literature search, among which twelve studies, including 625 patients, were included for analysis. The pooled prevalences of fecal incontinence, constipation, and bladder dysfunction symptoms and good to excellent bowel function scores were 0.20 (95% CI 0.13–0.28), 0.14 (95% CI 0.06–0.25), 0.07 (95% CI 0.04–0.12), and 0.95 (95% CI: 0.91–0.97), respectively; the pooled mean score of gastrointestinal-related quality of life was 118 (95% CI: 112.56–123.44).

**Conclusions:**

HD patients older than ten years old have an overall high prevalence of fecal incontinence and a low quality of life. Targeted and evidence-based follow-up procedures and transitional care are essential to meet these patients’ long-term care needs. Prospective and multicenter research that focuses on the attributes and predictors of the long-term prognosis of patients with HD are necessary.

## Background

New surgery techniques and enhanced recovery after surgery (ERAS) practice have improved the results after surgery for children with Hirschsprung disease (HD) by reducing the length of operation time, blood loss, use of analgesia and length of hospital stay [1–3]. Follow-up within the first three years after surgery shows that children who receive new surgical approaches have a lower onset of postoperative complications [4, 5]. Despite the encouraging short-term outcomes of these definitive surgeries, complications including constipation, fecal incontinence, and enterocolitis, among others, continue to burden some HD patients and jeopardize their quality of life (QoL) [6, 7]. To provide targeted interventions for these patients, identifying the prevalence of the prolonged postoperative complications and the characteristics of these patients is necessary.

Several meta-analysis have been conducted to compare the short- and mid-term postoperative outcomes among patients with HD who underwent different surgical approaches [8–12]; however, conclusions regarding the optimal surgical approach to obtain the best postoperative outcomes are conflicting, and the rates of complications are highly variable. To the best of our knowledge, no systematic review has been conducted on the prevalence of the long-term outcomes in children with HD surgical history who lived beyond their childhood.

The purpose of this study is to estimate the prevalences of fecal incontinence, constipation, bowel function, bladder dysfunction symptoms and QoL of patients with HD surgical history who reached ten years old or older. These findings can contribute to knowledge on the prognosis of patients with HD and facilitate the design of evidenced-based follow-up and better transitional care for these patients.

## Methods

We conducted a meta-analysis according to the review protocol (see Additional file 1) and Meta-analysis of Observational Studies in Epidemiology (MOOSE) guideline [13]. PubMed, AMED, Cochrane Library, CINAHL and PsycINFO databases were searched from inception to October 2018. To allow for a comprehensive literature search of all studies containing the long-term outcomes of patients with HD surgical history, no language or study design filters were used in our initial search. The search strategy used in PubMed was as follows: (“Hirschsprung Disease”[Mesh]) OR Mega colon) OR aganglionosis)) AND ((((((“Follow-Up Studies”[Mesh]) OR follow-up) OR (“Outcome and Process Assessment (Health Care)”[Mesh])) OR bowel function) OR “Quality of Life”[Mesh]) OR QoL), which was then adapted in line with the indexing systems of other databases. The reference lists of the included studies and existing systematic reviews were reviewed for additional relevant studies. Two key authors were contacted for further potential data relevant to this study.

### Selection criteria

**Fig. 1:**
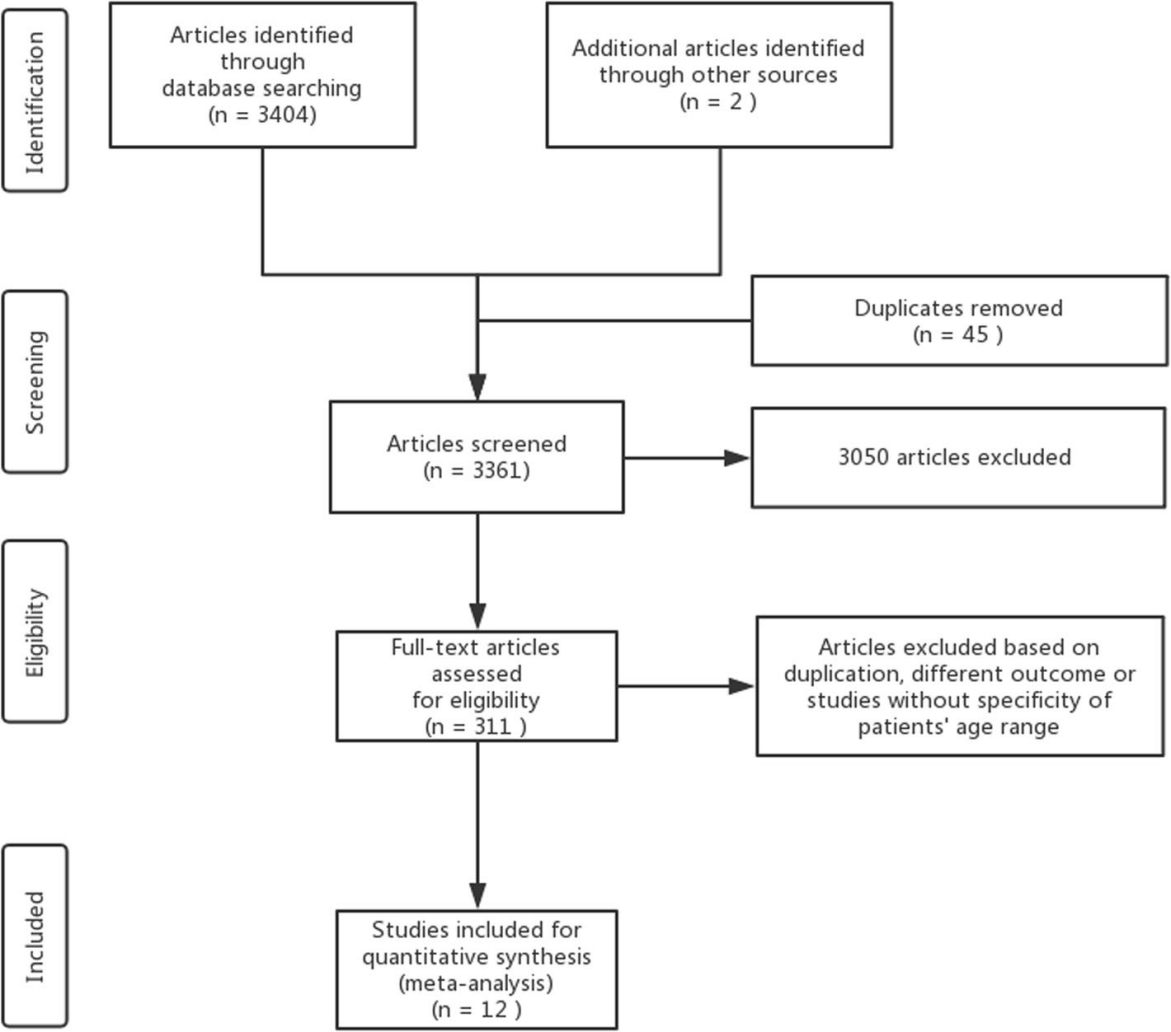
PRISMA flowchart of the screening process

Two reviewers independently scanned the titles and abstracts of the acquired articles for the initial screening. Original articles that reported the outcome or quality of life of patients older than ten years old with HD surgical history were included. The exclusion criteria were as follows: 1) non-English language papers, reviews, conference proceedings, and case reports or case series (fewer than 15 participants); 2) studies conducted on animal models or focused on analyzing the molecular biological or pathological mechanisms of HD; 3) studies focusing on parental stress and anxiety; and 4) studies including patients with a wider age range but without reporting the specific number of children older than ten years old. After the initial screening, the full text of 311 included articles was retrieved and read by two reviewers to determine their eligibility for inclusion in the analysis. The full screening process is listed in the PRISMA flowchart (see Fig. 1). Articles that were identified as potentially relevant in the initial screening and of which the full text was read are listed in Additional file 2.

### Data extraction

A standardized spreadsheet that included key characteristics, such as the study design, year of publication, geographical region, patients’ age range, category of HD (i.e., recto-sigmoid, long-segment, and total aganglionosis), fecal incontinence, constipation, bowel function score, bladder dysfunction symptoms and quality of life of patients with an HD surgical history, was developed for data collection. Two reviewers independently extracted information from the included articles. Discrepancies in the screening and data extraction process were discussed and resolved by consensus of the two reviewers.

### Definitions of related concepts

To differentiate similar soiling symptoms caused by functional constipation, we defined fecal incontinence based on the diagnostic criteria for non-retentive functional fecal incontinence as “uncontrolled loss of feces into places inappropriate to the social context, with no evidence of fecal retention” [14]. We used the definition of constipation that Diseth and colleagues defined in their study, namely, “having fewer than three defecations per week, or the need for regular laxatives, or both” [15]. Bladder dysfunction symptoms were defined by the International Children’s Continence Society (ICCS), including urinary incontinence (involuntary leakage of urine), sudden or unexpected urgent need to void, night enuresis (awakening to void at night), frequent urination (voiding eight or more times during waking hours), the need to apply abdominal pressure to initiate and maintain voiding, and burning or discomfort during voiding [16].

### Quality assessment

The quality of the included cohort and case-control studies was evaluated by the Newcastle-Ottawa Scale (NOS) [17], which contains three evaluation criteria: selection, comparability, and exposure. The total score of the NOS is 9, with a higher score indicating higher quality. A NOS score of 0–5, 5–6, and 7–9 was considered as low, medium and high quality respectively [18]. The Agency for Healthcare Research and Quality (AHRQ) checklist [19] was used to assess the quality of the included cross-sectional studies. The AHRQ checklist contains 11 items, with each item individually addresses a certain domain of the quality of the study and is answered with “yes”, “no”, or “unclear”, and does not incorporate an overall score (see Additional file 3).

### Statistical analysis

The “metaprop” command was employed to pool the prevalence of binary long-term outcomes (i.e., fecal incontinence, constipation, bladder dysfunction symptoms) [17]. The exact method was used to compute the specific confidence interval of each study. For continuous variables, including bowel function score and QoL score, the “metan” command was used to pool the mean differences. The heterogeneity and variation in the pooled estimations was computed by Cochrane’s Q test and the I^2^ test, respectively, with the *p* value < 0.05 considered statistically significant [18]. The pooled prevalence was calculated by the random effects model if the heterogeneity was higher than 25%; otherwise, the fixed effect model was employed. The sensitivity analysis was conducted by the sequential omission of individual studies with the “metaninf” command. A study was considered influential if the pooled mean estimate without it was not within the 95% CI bounds of the overall mean. Publication bias was evaluated by Egger’s linear regression test with the “metabias6” command, with the *p* value < 0.05 considered statistically significant [19, 20]. When the heterogeneity test showed I^2^ > 50%, the subgroup analysis was implemented [20]; this analysis was conducted for the geographical area, year of publication, patients’ age range, and age at surgery. Since most of the included studies did not report the specific number of complication events according to different categories of HD (i.e., the level of aganglionosis) or surgical technique, we were unable to perform subgroup analysis on these two factors. The whole procedure was conducted with Stata (version 14; Stata Corporation, College Station, TX).

## Results

### Study characteristics

The literature search identified 3406 potentially relevant articles (Fig. 1). After scanning the titles and abstracts, 311 articles were included for full-text screening to assess eligibility for inclusion. After full-text review, 12 articles with 625 patients older than ten years with an HD surgical history were included for analysis, among which four were case-control studies, six were cross-sectional studies, and the remaining two were cohort studies. These studies were implemented at various pediatric healthcare institutions in various countries, including Finland [21–24], Sweden [25, 26], Australia [27, 28], the UK [29], Canada [30], Japan [31], Norway [15] and Thailand [32]. Surgical approaches including the Duhamel and Soave methods were the most frequently reported approaches that were applied to these patients. Ten of the twelve studies included patients with congenital diseases or syndromes, with Down Syndrome being the most frequently reported. Most of the included cohort and case-control studies were evaluated as high or medium quality based on the NOS checklist, whereas most cross-sectional studies failed to mention whether the evaluators of subjective components of the study were masked to other aspects of participants’ status, nor do they describe any assessments undertaken for quality assurance (see Table 1 and Additional file 3).

**Table 1:** Characteristics and quality appraisal of included studies

Author	Year	Country	Study design	Age range of patients (years)	Number of patients older than 10 years	Rectosigmoid aganglionosis (n)	Long-segment (n)	Total colonic aganglionosis (n)	Surgery approach	Age at surgery (Mean, interquartile range), (year)	Associated congenital disease or syndrome (n)	Fecal incontinence (n)	Constipation (n)	Urinary system dysfunction (n)	Quality of appraisal (NOS/AHRQ checklist) ^**a**^
Granström et al. [25]	2015	Sweden	Case-control	20–43	39	37	/	2	Soave, Duhamel, Sphincteromyectomy, Ileostomy, Sigmoid colostomy.	1 (1, 17)	Down syndrome (n = 1), hypospadias (*n* = 2), congenital central hypoventilation syndrome (*n* = 1) and Fallot’s striad (n = 1)	/	/	3	S: 3, C: 1, E: 3 Total: 7
Jarvi et al. [21]	2010	Finland	Case-control	35–48	89	79	4	1	Soave, Duhamel, State Rehbein, Swenson, Colonal pull-through.	2.2 (0.8, 2.0)	Down syndrome (n = 1), cartilage hair hypoplasia (n = 1), and multiple endocrine neoplasia type 2 (n = 1).	12	27	/	S: 3, C: 1, E: 2 Total: 6
Neuvonen et al. [22]	2017	Finland	Case-control	18–32	33	66	7	3	Transanal endorectal pull-through (TEPT), TEPT with laparotomy/laparoscopy, Ileoanal pull-through; Definitive endostomy.	0.35 (0, 9.8)	Down syndrome (n = 11), Mowat-Wilson syndrome (*n* = 4), cartilage-hairhypoplasia (n = 2), Currarino syndrome (n = 1), Waardenburg-Shah syndrome (n = 1), and marker-chromosome syndrome (n = 1).	1	2	/	S: 4, C: 1, E: 2 Total: 7
Diseth et al. [15]	1997	Norway	Case-control	10–20	19	12	/	3	Duhamel	0.7 (0.1, 5)	Unclear	6	3	/	S: 3, C: 1, E: 3 Total: 7
Conway et al. [29]	2007	UK	Cohort study	16–23	78	63	15	0	Duhamel	0.75	Unclear	/	/	/	S: 2, C: 0, O: 2 Total: 4
Athanasakos et al. [27]	2006	Australia	Cross-sectional	13–24	23	50	8	14	Soave, Duhamel.	Unclear	Down syndrome (n = 2)	11	5	/	Yes: 6, No: 0, Unclear: 5
Heikkinen et al. 1995 & Heikkinen et al. 1997 [23, 24]	1995	Finland & UK	Cohort study	24–38	100	95	4	1	Duhamel, Swenson, State-Rehbein, Soave.	< 1 year old: 38, 1–3 years old: 37, older than 3 years old: 25	Down syndrome (n = 1), Chondroectodermal hypoplasia (n = 1), Marfan’s syndrome (n = 1).	14	1	/	S: 3, C: 1, O: 2 Total: 6
Mills et al. [30]	2008	Canada	Cross-sectional	13–18	16	34	10	3	Duhamel, Soave, Swenson, Transnal.	Unclear	Down syndrome (*n* = 3), cardiac anomalies (n = 2). Cleft palate (n = 1), ureteric reflux (n = 1), autism (n = 1), inguinal hernia (n = 1), congenital ptosis (n = 1), hypothyroidism (n = 1), and pyloric stenosis (n = 1),	1	1	0	Yes: 8, No: 1, Unclear: 2
Catto-Smith et al. [28]	2007	Australia	Cross-sectional	12–27	32	/	/	/	Soave, Duhamel, Swenson, Unknown.	Unclear	Down syndrome (*n* = 10)	7	18	4	Yes: 8, No: 1, Unclear: 2
Ieiri et al. [31]	2010	Japan	Cross-sectional	19–55	43	34	4	3	Z-shaped anastomosis (modified Duhamel procedure), Swenson, Martin.	Unclear	Unclear	15	2	5	Yes: 7, No: 1, Unclear: 3
Gunnarsdo’ttir et al. [4, 26]	2010	Sweden	Cross-sectional	18–45	42	29	8	3	Duhamel, Duhamel-Martin modification, Ileorectal anastomosis.	0.58 (0,11.6)	Down syndrome (*n* = 6)	13	5	2	Yes: 9, No: 0, Unclear: 2
Niramis et al. [32]	2008	Thailand	Cross-sectional	10–19	111	95	16	0	Duhamel, Soave, Swenson.	10–15 years old: 1 (0.5,13); older than 15 years old: 1.3 (0.6, 11)	Down syndrome (*n* = 9), cerebral palsy (n = 4) ^b^	17	11	/	Yes: 6, No: 2, Unclear: 3

^a^The NOS checklist was used to evaluate cohort studies and case-control studies, while the AHRQ checklist was used for cross-sectional studies. For cohort and case-control study, the total score of the NOS is 9, with higher score indicating higher-quality. S: Selection, C: Comparability, E: Exposure, O: Outcome. Score lower than 5 was considered low, 5–6 medium, and 7–9 high quality. For cross-sectional study, the overall quality of reports of the included studies was marginal to fair (Additional file 3). For example, most of the studies failed to report whether the patients were consecutively enrolled, which could be subjective to selection bias

^b^In this study, patients with associated malformations were excluded for statistical analysis

### Fecal incontinence

**Fig. 2:**
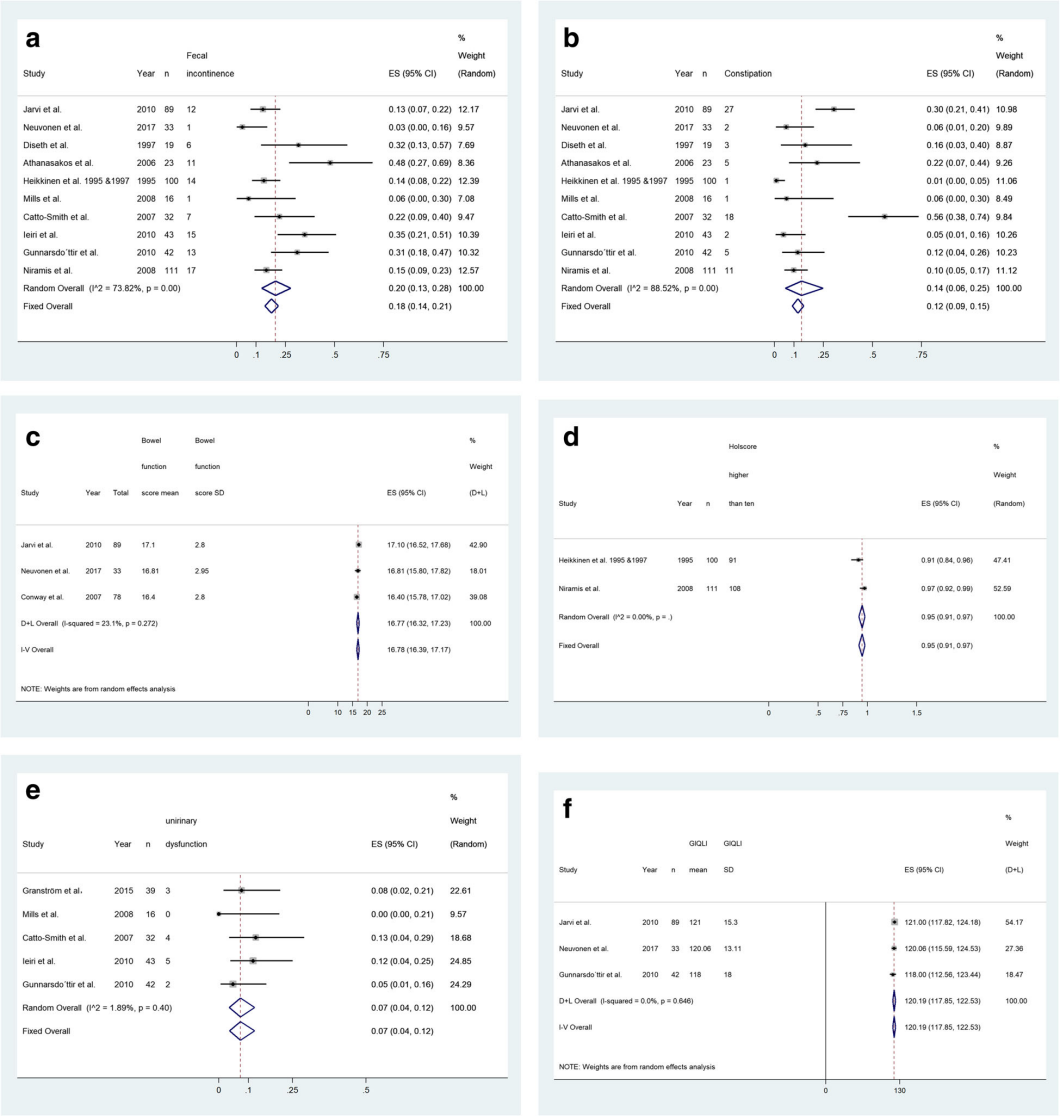
**a**. Pooled prevalence of fecal incontinence. **b**. Pooled prevalence of constipation. **c**. Pooled mean score of Bowel Function Score (BFS score). **d**. Pooled prevalence of patients with excellent to good Holschneider Score. **e**. Pooled prevalence of patients with bladder dysfunction symptom. **f**. Pooled mean score of gastrointestinal quality of life index score (GIQLI score)

A total of 508 patients were evaluated for long-term fecal incontinence. The pooled estimation of the prevalence of fecal incontinence is 0.20 (95% CI, 0.13–0.28, Cochran Q test, *P* <  0.001, I^2^ = 73.82%, see Fig. 2a), with the largest study (*n* = 111) reporting a fecal incontinence prevalence of 0.15 (95% CI: 0.09, 0.23) [32].

### Constipation

A total of 508 patients underwent a constipation evaluation. The overall pooled prevalence of constipation is 0.14 (95% CI: 0.06–0.25, Cochran Q test, P <  0.001, I^2^ = 88.52%, see Fig. 2b).

### Bowel function score

Six studies reported bowel function scores in a total of 411 participants, with four studies [21, 22, 29, 33] using the Bowel Function Score (BFS) [34] and the remaining two studies [23, 32] using the Holschneider scale [35]. The BFS contains seven items, with each item scored from 0 to 3, except for one item scored from 1 to 2 (see Table 2). The whole score of BFS is 20, and a score ≥ 17 is defined as the lower limit of a normal bowel function [36]. Since one study [33] only reported participants’ mean BFS score from each domain without reporting the overall score, this study was not included in the analysis of bowel function. The pooled overall mean BFS score is 16.78 (95% CI: 16.34–17.17, Cochran Q test, *P* = 0.27, I^2^ = 23.1%, see Fig. 2c), and the prevalence of patients with excellent to good Holschneider scores (i.e., score > 10) is 0.95 (95% CI: 0.91–0.97, I^2^ = 0, see Fig. 2d).

**Table 2:** Bowel Function Score

Factor	Score Given
Ability to hold back defecation	
Always	3
Problems < 1/week	2
Weekly problems	1
No voluntary control	0
Feels the urge to defecate	
Always	3
Most of the time	2
Uncertain	1
Absent	0
Frequency of defecation	
Every other day to twice a day	2
More often	1
Less often	1
Soiling	
Never	3
Staining < 1/week, no change of underwear required	2
Frequently staining/soiling, change of underwear required	1
Daily soiling, requires protective aids	0
Accidents	
Never	3
Less than 1/week	2
Weekly accidents, often requires protective aids	1
Daily, protective aids required day and night	0
Constipation	
No constipation	3
Manageable with diet	2
Manageable with laxatives	1
Manageable with enemas	0
Social problems	
No social problems	3
Sometimes (foul odors)	2
Problems causing restrictions of social life	1
Major social/psychological problems	0

### Bladder dysfunction symptoms

Urinary system function was assessed in 172 patients. The overall prevalence of bladder dysfunction symptoms is 0.07 (95% CI: 0.04–0.12, Cochran Q test, *P* = 0.40, I^2^ = 1.89%, see Fig. 2e), with the largest study (*n* = 43) reporting a prevalence rate of 0.12 (95% CI: 0.04, 0.25) [31]. Compared to studies that only included adult patients (> 18 years old), Mills et al. included younger patients aged between 13 and 18 years and reported no bladder dysfunction symptoms among these patients [30].

### Gastrointestinal quality of life

Three studies [21, 22, 26] reported HD patients’ gastrointestinal quality of life index (GIQLI) with the mean and standard deviation. The GIQLI is a validated scale that evaluates the quality of life of patients with gastrointestinal issues from 36 items, with the total score of 144, and a score of 125.8 (95% CI 121.5–127.5) as the average score of the general population [21, 37]. The pooled estimation of the mean GIQLI is 120.19 (95% CI: 117.85–122.53, Cochran Q test, *P* = 0.65, I^2^ = 0, see Fig. 2f). The mean GIQLI score for the study with the largest sample (*n* = 89) is 121 (95% CI 117.82–124.18) [21]. The lowest mean GIQLI score is 118 (95% CI: 112.56–123.44) [26], which is higher than the cutoff score (i.e., score of 105) for constant gastrointestinal symptoms [37].

### Subgroup analysis

Subgroup analysis of the prevalence of fecal incontinence, constipation and bladder dysfunction were conducted for the following categories: year of publication (before or after 2008), geographic region (Europe, North America, Oceania, or Asia), study design (case control, cohort, or cross-sectional), patient age at follow-up (younger or older than 19 years), and age at surgery (younger or older than 0.5 years). Because of the lack of sufficient data, we were unable to conduct subgroup analysis for three categories that could potentially influence heterogeneity: type of surgery, patients’ length of aganglionic colonic segment, and patients with associated congenital diseases or symptoms.

Although no clinical heterogeneity was found to be caused by the above categories, participants from the Oceania area [27] have the highest prevalence of fecal incontinence, constipation and bladder dysfunction, with pooled prevalences of 0.33, 0.42, and 0.13, respectively. Regarding study design, cross-sectional and case-control studies have the highest prevalence of fecal incontinence and constipation, respectively. Patients who received surgery later than five months of age and patients who had lived to 19 years or older when they received follow-up have higher prevalences of fecal incontinence, constipation and bladder dysfunction (see Table 3).

**Table 3:** Fecal incontinence subgroup analysis for year of publication, geographic region, study design and age range

Long-term outcome	Subgroup characteristics	Subgroup categories	Number of studies	Pooled prevalence / mean (95% CI)	Heterogeneity (I^**2**^, ***P***)
Fecal incontinence	Year of publication	< 2008	6	0.19 (0.07, 0.30)	0, 0.71
		> 2008	4	0.20 (0.06, 0.33)	0, 0.42
	Geographic region	Europe	5	0.16 (0.05, 0.28)	0, 0.72
		Oceania	2	0.33 (0.07, 0.59)	0, 0.34
		Asia	2	0.21 (0.04, 0.40)	16.5%, 0.27
		North America	1	0.06 (−0.42, 0.55)	N/A
	Study design	Case-control	3	0.14 (−0.03, 0.30)	0, 0.61
		Cohort study	1	0.14 (− 0.06, 0.34)	N/A
		Cross-sectional	6	0.24 (0.12, 0.36)	0, 0.63
	Age at follow-up (years) ^a^	≤ 19	2	0.14 (−0.03, 0.32)	0, 0.73
		> 20	4	0.20 (0.08, 0.32)	0, 0.53
	Age at surgery (years) ^b^	< 0.5	1	0.03 (−0.31, 0.37)	N/A
		≥ 0.5	5	0.17 (0.07, 0.28)	0, 0.83
Constipation	Year of publication	< 2008	6	0.16 (0.02, 0.31)	36.7%, 0.162
		> 2008	4	0.17 (0.04, 0.31)	0, 0.43
	Geographic region	Europe	5	0.13 (0.02, 0.25)	7.3%, 0.37
		Oceania	2	0.42 (0.16, 0.68)	38.4%, 0.20
		Asia	2	0.08 (−0.07, 0.24)	0, 0.77
		North America	1	0.06 (− 0.42, 0.55)	N/A
	Study design	Case-control	3	0.23 (0.06, 0.39)	0, 0.46
		Cohort study	1	0.01 (−0.19, 0.21)	N/A
		Cross-sectional	6	0.17 (0.02, 0.31)	23.6%, 0.257
	Age at follow-up (years) ^a^	≤ 19	2	0.09 (−0.08, 0.27)	0, 0.89
		> 20	4	0.13 (0.01, 0.25)	32.5%, 0.22
	Age at surgery (years) ^b^	< 0.5	1	0.06 (−0.28, 0.40)	N/A
		≥ 0.5	5	0.13 (0.03, 0.23)	6.2%, 0.37
Bladder dysfunction	Year of publication	< 2008	2	0.08 (−0.20, 0.36)	0, 0.68
		> 2008	3	0.08 (−0.09, 0.26)	0, 0.95
	Geographic region	Europe	2	0.06 (−0.16, 0.28)	0, 0.90
		Oceania	1	0.13 (−0.22, 0.47)	N/A
		Asia	1	0.12 (−0.18, 0.41)	N/A
		North America	1	0.00 (−0.48, 0.48)	N/A
	Study design	Case-control	1	0.08 (−0.24, 0.39)	N/A
		Cohort study	0	N/A	N/A
		Cross-sectional	4	0.08 (−0.09, 0.25)	23.6%, 0.257
	Age at follow-up (years) ^a^	≤ 19	1	0.00 (−0.48, 0.48)	N/A
		> 20	3	0.08 (−0.09, 0.26)	0, 0.95
	Age at surgery (years) ^b^	< 0.5	2	0.06 (−0.16, 0.28)	0, 0.90
		Unclear	3	0.10 (−0.11, 0.30)	0, 0.91

^a^Only studies with all participants older than 20 years or younger than 19 years were included

^b^Only studies reporting the patients’ age when they received surgery were included

### Sensitivity and publication bias

**Fig. 3:**
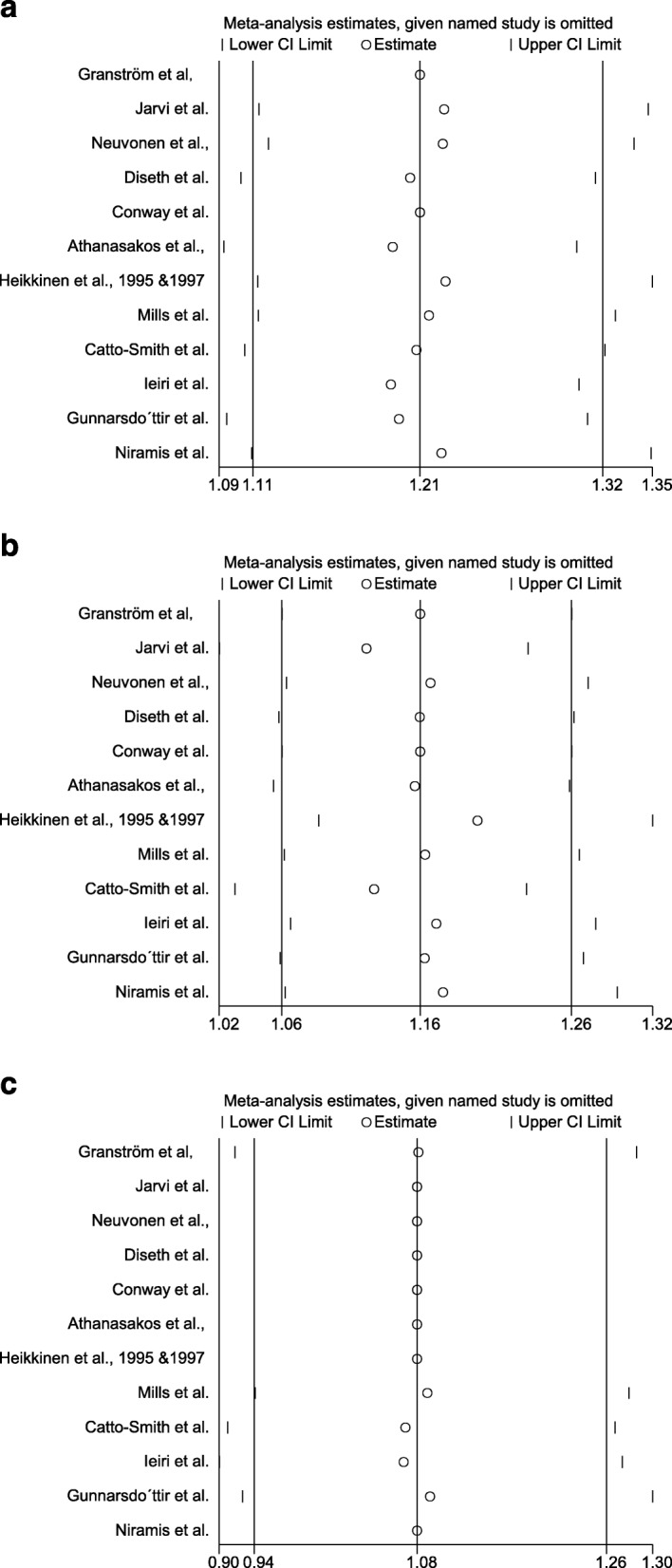
**a**. Sensitivity analysis of combined relative frequency of patients with fecal incontinence. **b**. Sensitivity analysis of combined relative frequency of patients with constipation. **c**. Sensitivity analysis of combined relative frequency of patients with bladder dysfunction symptom

The sensitivity analysis from sequential omission of the long-term fecal incontinence, constipation, and bladder dysfunction symptom was listed in Fig. 3a, b, and c, respectively, suggesting that the combined relative frequency was not altered after omission. Egger’s regression test showed that the *p* values of publication bias for the prevalences of fecal incontinence, constipation, bladder dysfunction symptoms, BFS score, Holschneider score, and GIGLI score are 0.23, 0.61, 0.37, 0.95, 0.32, and 0.19, respectively, suggesting that no publication bias is found. Egger’s publication bias graphs are listed in Additional file 4.

## Discussion

In this study we estimate that for patients older than ten years with an HD surgical history, the prevalences of fecal incontinence, constipation and bladder dysfunction symptoms are 20, 14 and 7%, respectively; and these patients generally have lower gastrointestinal quality of life index compared to healthy population.

### Comparison with other literature

The pooled prevalence of fecal incontinence in patients with HD who were older than ten years is 20% (95% CI 0.13–0.28), which is much higher than that of the general population (1.6% in teenagers and 7.7% in adults) [14, 38]. In other studies that focus on patients with HD, the reported prevalence of fecal incontinence ranges from 9.8 to 37.8% [39–42]. Discrepancies among these studies may be due to the heterogeneity in geographical regions, sample size, participants’ characteristics, definition of fecal incontinence, and follow-up period of the patients.

Regarding the epidemiology of constipation, we estimated that 14% (95% CI: 0.06–0.25) of patients with HD experience constipation onset when they reach ten years old, which is comparable to the estimate of the prevalence of constipation in the general population (16% in teenagers and 12 to 19% in adults) [43, 44], but slightly lower than that of another study [45] in which 25% of patients with HD were reported to have constipation. Chung et al. reported a constipation prevalence of 17.5% in patients with short-segment HD after 52 months of definitive surgery [46]. Another study employed the Krickenbeck criteria to diagnose constipation and reported a constipation prevalence of 25% [47]. Reasons for the heterogeneity in the prevalence of constipation among different studies may be similar to those for fecal incontinence heterogeneity.

Only a small number of participants (*n* = 127) were included in the bladder dysfunction symptoms analysis. The overall prevalence of bladder dysfunction symptoms is 0.07 (95% CI: 0.04–0.12), which is slightly higher than the prevalences of urinary urgency, daytime incontinence, emptying difficulties or enuresis in children aged 17 [16]. Xiong et al. reported no occurrence of urinary retention in their cohort of Chinese patients with HD [33], suggesting that patients generally have satisfactory urinary function.

Previous studies have been inconsistent in concluding whether the long-term outcomes for HD patients improve over time. In this study, teenagers have slightly lower prevalences of constipation, fecal incontinence and bladder dysfunction, although this variance is not statistically significant. Meinds et al. reported significant improvements in fecal incontinence in the adult group compared to the children’s group (16.8 versus 37.6%, *p* <  0.001), but constipation remained constant (22.0% in both groups) [42]. Granström and colleagues reported that the occurrence of constipation decreased from 41 to 14% during a three-year follow-up period, but the prevalence of fecal incontinence remained high among the same patient group (ranged from 56 to 67%) [48]. Another study founded that fecal incontinence improved over time – particularly when children reach late adolescence [49]. Since some of these original studies are limited in their small sample size, moderate rate of loss to follow-up [50], and potential bias in that the person who evaluated the patients’ outcomes was not blinded to the patients’ disease and surgery information, it is difficult to draw conclusions regarding changes in the long-term outcomes of patients with HD. Prospective multicenter studies of these patients’ health-related outcomes that involve longer and more standardized follow-up and thorough research designs are necessary.

All the included original studies in this meta-analysis employed either the Bowel Function Score (BFS) or the Holschneider score to evaluate patients’ bowel function. The pooled overall mean BFS score is 16.78 (95% CI: 16.34–17.17); this score marginally reaches the cutoff value of good/normal bowel function (BFS > 17), while the pooled prevalence of patients with excellent to good Holschneider scores (i.e., score > 10) was 0.95 (95% CI: 0.91–0.97). A study of the long-term outcomes of a cohort of 200 Nordic patients with HD also reported BFS scores for children aged 13–17 years and children older than 18 years of 18 (8–20) and 19 (13–20), respectively [45]; these values are slightly higher than the results of this study. The pooled mean bowel function scores in this meta-analysis and in the results from other literature suggest that most patients older than ten years with an HD surgical history tend to have good to excellent bowel function, despite different tools being used to evaluate the bowel function of these patients.

The pooled mean GIQLI is 120.19 (95% CI: 117.85–122.53), which is higher than the cutoff score (i.e., 105) of constant gastrointestinal symptoms [22]; however, it was lower than the average score of the general population (125.8, 95% CI 121.5–127.5) [37], suggesting that although most patients do not have consistent gastrointestinal symptoms, their health-related quality of life is not as good as their healthy peers. Hartman and colleagues found in their literature review that HD patients could have a good quality of life while experiencing worse disease-specific functioning [51]. The inconsistency between bowel function and quality of life may be due to the psychological symptoms and feelings, such as anxiety and unhappiness caused by HD, which can have a substantial negative influence on these patients’ quality of life. Another possible reason is that parental stress and parental self-efficacy are associated with children’s health outcomes [52, 53], and may even play a mediating role between children’s health-related behavior and quality of life [54]. Two of the included studies [22, 30] employed the PedQoL 4.0 inventory to evaluate children’s quality of life; however, since only one study reported the results of children older than twelve, we were unable to pool the mean scores for quality of life for adolescent patients and to explore how quality of life changes from adolescence to adulthood.

### Strengths and limitations

This study is limited in its small number of participants in the included original studies, and the quality of most of the included cross-sectional studies was marginal to fair; thus, the conclusions of this study should be interpreted with caution. Only two studies employed a prospective cohort design, with heterogeneous contents of long-term outcomes and duration and interval of follow-up periods, which makes comparisons between the outcomes of these two patient cohorts difficult. Additionally, most of the included studies failed to report the long-term outcomes from the stratification of the length of aganglionosis colonic bowel segment, type of surgical procedure, and associated congenital diseases, which may influence the prognosis of patients with HD. Another limitation is that only English papers were included in this study; thus, the results may be subject to a language bias. Despite these limitations, this meta-analysis conducted a comprehensive literature search, included the latest evidence, and employed a rigorous statistical method to integrate the pooled prevalence of the long-term prognosis of patients with HD with greater statistical power (by enlarging the whole sample size) [55, 56].

### Implications for future research

Providing accurate estimations of the long-term prognosis of patients with HD, recognizing those at a high risk of poor outcomes, and providing these patients with targeted transitional care is of great importance for better recovery and quality of life for the whole group of patients with HD. Future research should involve multicenter studies with standardized outcome indicators, follow-up durations and intervals and should help design evidence-based transitional care for these patients. A more in-depth analysis of the prognosis of patients with HD from the stratification of the length of aganglionosis bowel segment, patients’ age and gender, and surgical procedure is also necessary.

## Conclusions

Compared to the general population, adolescent and adult patients with HD surgical history tend to have a higher prevalence of fecal incontinence and lower gastrointestinal-related quality of life, although these patients generally have satisfactory bowel and urinary function. Healthcare professionals should pay closer attention to the long-term prognosis of patients with HD and provide those at a high risk of poor outcomes with more proactive transitional care.

## Supplementary information

**Additional file 1.**

**Additional file 2.** Reference list of potentially relevant articles that were read in full-text form.

**Additional file 3.** Quality appraisal of cross-sectional studies.

**Additional file 4.**

## Data Availability

All data generated or analyzed during this study are included in this article.

## Abbreviations

AHRQ: The Agency for Healthcare Research and Quality
GIQLI: Gastrointestinal Quality of Life Index
HD: Hirschsprung disease
ICCS: The International Children’s Continence Society
MOOSE: Meta-analysis of Observational Studies in Epidemiology
NOS: The Newcastle-Ottawa Scale
PedQoL: Pediatric Quality of Life Inventory
QoL: Quality of Life
