# Supplementary material for: Long-term outcomes and quality of life of patients with Hirschsprung disease: a systematic review and meta-analysis
Source: BMC Gastroenterol. 2020 Mar 12;20:67. doi: 10.1186/s12876-020-01208-z (PMC7066788; doi:10.1186/s12876-020-01208-z)
Supplement: Supplementary file 1 — Additional file 1. [file 12876_2020_1208_MOESM1_ESM.docx]

**Long-term Outcome and Quality of Life of Patients with Hirschsprung’s Disease: A Systematic Review and Meta-analysis Protocol**

**Ying Dai^1^, Yongfang Deng^2^, Yan Lin^3^, Runxian Ouyang^1^, Le Li^1*^**

1. Department of Pediatric Surgery, Guangzhou Women and Children’s Medical Center, Guangzhou Medical University, Guangzhou, China.

2. Department of Obstetrics and Gynecology, Guangzhou Women and Children's Medical Center, Guangzhou Medical University, Guangzhou, China.

3. Department of Nursing, Guangzhou Women and Children's Medical Center, Guangzhou Medical University, No. 9 Jinsui Road, Guangzhou, China

* Corresponding author (Contacting email: [Doctorlile@aliyun.com](mailto:Doctorlile@aliyun.com)).

**Abstract**

**Introduction:** Despite the advancement of surgery technique and perioperative care, it is still common for patients with Hirschsprung disease (HD) to have morbidities after the definitive surgery. Some patients have to endure long-lasting bowel and genitourinary dysfunction, which can negatively affect their quality of life and fuel the transitional and long-term care burden of these patients. This systematic review aims to evaluate the global prevalence of the long-term postoperative prognosis of patients with HD.

**Methods and analysis**: This review will follow the Meta-analysis of Observational Studies in Epidemiology (MOOSE) guideline, and include observational that involved patients diagnosed with HD and older than ten years and reported the prevalence of their post-operative prognosis and quality of life. Electronic databases including PubMed, AMED, Cochrane Library, CINAHL, and PsycINFO will be searched from inception to October 2018, followed by screening the reference lists of included studies and existing systematic reviews. Key author(s) will be contacted for relevant research and potential unpublished data. Inclusion criteria will be original observational studies that reported the postoperative outcome and quality of life of patients with HD and older than ten years. A standardized spreadsheet will be developed and piloted to extract relevant data. Two investigators will independently screen, select eligible studies and extract data. The Newcastle-Ottawa Scale will be employed to evaluate the quality of included cohort and case control studies, while the Agency for Healthcare Research and Quality checklist will be used to assess the quality of included cross-sectional studies. The prevalences of patients’ post-operative prognosis will be pooled by the “metaprop” and “metan” command contained in Stata (version 14), and standard mean differences will be calculated for continuous variables. Heterogeneity and variation will be pooled by Cochrane’s Q test and I^2^ respectively. Publication bias will be evaluated by Egger’s linear regression test. Sub-group analysis will be conducted when heterogeneity test I^2^ > 50%.

**Ethics and dissemination**: This review wholly rely on published data. Thus an ethical approval is not required. This study is expected to provide new evidence on the global prevalence and characteristics of the long-term prognosis and quality of life of patients with HD. The results will be disseminated by publication of the manuscript in a peer-reviewed journal.

**Background**

Hirschsprung disease (HD), characterized by the dearth of ganglion cells in distal bowel, is one of the most common congenital intestinal malformations and could be life-threatening for neonates and children if left untreated.[1] To date surgery is still the principal treatment for HD.

New surgical techniques and enhanced recovery after surgery (ERAS) practice have improved the effect of surgery treatment of children with Hirschsprung disease (HD), reducing the length of operation time, children’s blood loss, use of analgesia and length of hospital stay [2-4]. Follow-up within the first three years after surgery shows that children who receive new surgery approaches have lower onset of postoperative complications [5, 6]. Despite the encouraging short-term outcome of these definitive surgery, complications including constipation, fecal incontinence, enterocolitis, among others, continue to burden some HD patients and jeopardize their quality of life [7, 8]. To provide targeted intervention to these patients, identifying the prevalence of the postoperative complication and characteristics of these patients is needed.

Several meta-analysis have been conducted to compare the short- and mid-term postoperative outcome among different surgery approaches, yet their conclusions of the optimal surgery approach to reach best operative outcome are conflicting, and the rates of complications are highly variable [9-13]. To the best of our knowledge, no systematic review have been conducted on the prevalence of the long-term outcome of children with HD surgery history and reached beyond their childhood.

The purpose of this study is to estimate the prevalence of fecal incontinence, constipation, bowel function, bladder dysfunction symptoms and QoL of patients with HD surgery history that reached ten year old, which could contribute to the knowledge of the prognosis of patients with HD, and facilitate the design of evidenced-based follow-up and transitional care for them.

**Methods**

***Data sources and search strategy***

The study will follow the Meta-analysis of Observational Studies in Epidemiology (MOOSE) guideline [14]. Databases including PubMed, AMED, Cochrane Library, CINAHL and PsycINFO will be searched from inception to present. The search strategy will include both subject terms and free-text terms, which will be combined with the Boolean operator (i.e. “OR”, “AND”, “NOT”). The medical subject headings (MeSH) terms will include "Hirschsprung Disease", "Follow-up studies", "Outcome and Process Assessment (Health Care) ", and "Quality of life". All MeSH terms will be exploded to decrease the chance of missing important literature. The search strategy of PubMed was: ("Hirschsprung Disease"[Mesh]) OR Mega colon) OR aganglionosis)) AND (((((("Follow-Up Studies"[Mesh]) OR follow-up) OR ("Outcome and Process Assessment (Health Care)"[Mesh])) OR bowel function) OR "Quality of Life"[Mesh]) OR QoL). The search strategy will be adapted in line with the indexing systems of other databases.
No language or study design filter will be used in initial search to enhance the comprehensibility of the literature search. Reference lists of included studies and existing systematic reviews will be screened for additional relevant studies. Key authors will be contacted for further potential data relevant to this study.

***Inclusion criteria***

Observational studies, including cross-sectional, cohort and case-control studies that report the postoperative outcomes of patients with HD and reached ten year old will be included. Multiple papers that generated from the same data source will be reviewed and only relevant data will be included.

***Exclusion criteria***

Studies that have the following characteristics will be excluded:

1) Non-English language papers, reviews, conference proceedings, and case reports or case series (number of participants less than 15);

2) Studies conducted on animal models or focus on analyzing the molecular biological or pathological mechanism of HD;

3) Studies focusing on parental stress and anxiety;

4) Studies include patients with wider age range but without reporting the specific number of children older than ten years old.

***Screening strategy***

Assessment for eligibility of retrieved paper will be independently conducted by two authors. The screening include the following two phases. First, during the initial screening two authors will scan the title and abstract of studies. For papers that cannot be determined from their title and abstract, full-text will be retrieved and read to determine their eligibility. Disagreements between the two authors will be resolved by discussion.

***Data extraction***

A standardized data extraction form will be developed and piloted. The form will include year, country, study design, patients’ age and gender range, number of patients responded and the number of patients included for analysis, category of HD, type of surgery, age at surgery, age at follow-up, associated malformation, complication happened after surgery (i.e. fecal incontinence, constipation, enterocolitis, urinary dysfunction), and quality of life. If essential data are missing, contact to the authors of the study will be made for further information. Two authors will independently extracted information from each study. Discrepancies will solved by consensus.

***Data management***

The results of literature search will be stored in Endnote, and data extraction forms will be uploaded to the Guangzhou Women and Children’s Medical Center staff-allocated net work storage, which contains password and only accessible to the authors of this review. The shared network will facilitate data extraction and keep record of all review-related documents.

***Quality assessment***

The quality of cohort and case-control studies will be evaluated by the Newcastle-Ottawa Scale (NOS) [15], while cross-sectional studies will be assessed by the Agency for Healthcare Research and Quality (AHRQ) checklist [16].

***Statistical analysis***

The statistical analysis will conducted with Stata (version 14; Stata Corporation, College Station, TX). The “metaprop” command will be employed to pool the prevalence of binary long-term outcome (i.e. fecal incontinence, constipation, urinary system dysfunction) [17]. The exact method will be used to compute the specific confidence interval of each study. For continuous variables including bowel function score and QoL score, the “metan” command will be used to pool the mean differences. Heterogeneity and the variation in the pooled estimations will be computed by Cochrane’s Q test and I^2^ respectively, with p value < 0.05 considered statically significant [18]. The pooled prevalence will be calculated by the random effect model if heterogeneity is higher than 25%, otherwise the fixed effect model will be employed. Sensitivity analysis was conducted by sequential omission of individual studies with the “metaninf” command. A study will be considered to be influential if the pooled mean estimate without it is not within the 95% CI bounds of the overall mean. Publication bias will be evaluated by Egger’s linear regression test with the “metabias6” command, with p value < 0.05 were considered statistically significant [19, 20]. When heterogeneity test shows I^2^ > 50%, sub-group analysis will implemented on the geographical area, year of publication, patients’ age range and category of HD.

**Results reporting and presentation**

The proposed systematic review will be reported following the MOOSE guideline. The screening process will be described with the Preferred Reporting Items for Systematic Review and Meta-Analysis Protocols flow diagram. Tables and forest plots will be displayed to summarise quantitative data. The characteristic and quality of included studies will be narratively described in the manuscript and displayed in a table.

**Conclusion**

This systematic review and meta-analysis will provide pooled prevalence estimates of postoperative complications of patients with HD and reached ten years old. The comprehensive and rigorous meta analysis technique employed in this study will provide a robust knowledge synthesis of available data. We expect to provide accurate evidence for effective policy making. This study may identify existing research gaps to form the basis of future research to improve our understanding of the long-term prevalence of prognosis of HD and targeted intervention to improve remaining complications and patients’ quality of life.

**References:**

1. Puri P, Friedmatcher F: **Hirschsprung's disease**. In: *Rickham's Neonatal Surgery.* edn. London: Springer; 2018: 809-828.

2. Deng X, Wu Y, Zeng L, Zhang J, Zhou J, Qiu R: **Comparative Analysis of Modified Laparoscopic Swenson and Laparoscopic Soave Procedure for Short-Segment Hirschsprung Disease in Children**. *Eur J Pediatr Surg* 2015, **25**(5):430-434.

3. Xia X, Li N, Wei J, Zhang W, Yu D, Zhu T, Feng J: **Laparoscopy-assisted versus transabdominal reoperation in Hirschprung's disease for residual aganglionosis and transition zone pathology after transanal pull-through**. *J Pediatr Surg* 2016, **51**(4):577-581.

4. Ademuyiwa AO, Bode CO, Idiodi-Thomas HO, Elebute OA: **Early outcome of open primary pull through versus staged pull through in Hirschsprung's disease: a single centre experience from Nigeria**. *Nig Q J Hosp Med* 2012, **22**(3):164-167.

5. Gunnarsdottir A, Larsson LT, Arnbjornsson E: **Transanal endorectal vs. Duhamel pull-through for Hirschsprung's disease**. *Eur J Pediatr Surg* 2010, **20**(4):242-246.

6. Lukac M, Antunovic SS, Vujovic D, Petronic I, Nikolic D, Radlovic V, Krstajic T, Krstic Z: **Effectiveness of various surgical methods in treatment of Hirschsprung's disease in children**. *Vojnosanitetski pregled* 2016, **73**(3):246-250.

7. Collins L, Collis B, Trajanovska M, Khanal R, Hutson JM, Teague WJ, King SK: **Quality of life outcomes in children with Hirschsprung disease**. *J Pediatr Surg* 2017, **52**(12):2006-2010.

8. Engum SA, Grosfeld JL: **Long-term results of treatment of Hirschsprung's disease**. *Semin Pediatr Surg* 2004, **13**(4):273-285.

9. Zimmer J, Tomuschat C, Puri P: **Long-term results of transanal pull-through for Hirschsprung's disease: a meta-analysis**. *Pediatr Surg Int* 2016, **32**(8):743-749.

10. Shisong Z, Juan L, Yurui W, Yuanjun H, Chunhong D, Meiyun W, Zhongtao G, Zhang S, Li J, Wu Y *et al*: **Comparison of Laparoscopic-Assisted Operations and Laparotomy Operations for the Treatment of Hirschsprung Disease**. *Medicine* 2015.

11. Shogo S, Hiromu M, Hock A, Yuhki K, Chen Y, Lee C, Bo L, Pierro A, Seo S, Miyake H *et al*: **Duhamel and Transanal Endorectal Pull-throughs for Hirschsprung’ Disease: A Systematic Review and Meta-analysis**. 2017.

12. Thomson D, Allin B, Long AM, Bradnock T, Walker G, Knight M: **Laparoscopic assistance for primary transanal pull-through in Hirschsprung's disease: a systematic review and meta-analysis**. *BMJ open* 2015, **5**(3):e006063.

13. Guerra J, Wayne C, Musambe T, Nasr A: **Laparoscopic-assisted transanal pull-through (LATP) versus complete transanal pull-through (CTP) in the surgical management of Hirschsprung's disease**. *J Pediatr Surg* 2016, **51**(5):770-774.

14. Stroup DFB, Jesse A.; Morton, Sally C.; et al.: **Meta-analysis of Observational Studies in Epidemiology: A Proposal for Reporting**. *JAMA* 2000, **283**(15).

15. Wells G, Shea B, O’connell D, Peterson J, Welch V, Losos M, Tugwell P: **The Newcastle-Ottawa Scale (NOS) for assessing the quality of nonrandomised studies in meta-analyses. Ottawa Hospital Research Institute, 2014**. In*.*: oxford. ASp; 2015.

16. Rostom A, Dubé C, Cranney A, Saloojee N, Sy R, Garritty C, Sampson M, Zhang L, Yazdi F, Mamaladze V *et al*: **Celiac Disease. Evidence Report/Technology Assessment No. 104. (Prepared by the University of Ottawa Evidence-based Practice Center, under Contract No. 290-02-0021.)**. In*.*: AHRQ Publication No. 04-E029-2. Rockville, MD: Agency for Healthcare Research and Quality; 2004.

17. N NV, Marc A, Marc A: **Metaprop: a Stata command to perform meta-analysis of binomial data**. *Archives ofPublic Health* 2014, **72**:39.

18. Huedo-Medina TB, Sánchez-Meca J, Marín-Martínez F, Botella J: **Assessing heterogeneity in meta-analysis: Q statistic or I² index?** *Psychological methods* 2006, **11**(2):193.

19. Egger M, Smith GD, Schneider M, Minder C: **Bias in meta-analysis detected by a simple, graphical test**. *Bmj* 1997, **315**(7109):629-634.

20. Zhang T, Dong S, Zhou Z: **Advanced Meta-analysis in Stata**: Fudanpress; 2015.
