# Supplementary material for: Long-term outcomes and quality of life of patients with Hirschsprung disease: a systematic review and meta-analysis
Source: BMC Gastroenterol. 2020 Mar 12;20:67. doi: 10.1186/s12876-020-01208-z (PMC7066788; doi:10.1186/s12876-020-01208-z)
Supplement: Supplementary file 2 — Additional file 2. Reference list of potentially relevant articles that were read in full-text form. [file 12876_2020_1208_MOESM2_ESM.docx]

Additional file 2. Reference list of potentially relevant articles that were read in full-text form.

[1-150][151-311]

1. Ateş U, Bahadır K, Göllü G, Taştekin NY, Ergün E, Bingöl-Koloğlu M, Çakmak M, Aktuğ T, Dindar H, Yağmurlu A: **Hirschsprung Hastalığında Laparoskopik Seviyelendirmenin Tedavi ve Yönetime Katkısı [Contrubition of Laparoscopic Level Determination on Hirschsprung Disease Diagnosis and Management]**. *Med Bull Haseki* 2018, **56**:218-221.

2. Chaeyoun O, Sanghoon L, Suk-Koo L, Jeong-Meen S, Oh C, Lee S, Lee S-K, Seo J-M: **Difference of Postoperative Stool Frequency in Hirschsprung Disease According to Anastomosis Level in a Single-Stage, Laparoscopy-Assisted Transanal Endorectal Pull-Through Procedure**. *Medicine (Baltimore)* 2016, **95**(14):e3092.

3. Huang W-K, Li X-L, Zhang J, Zhang S-C: **Prevalence, Risk Factors, and Prognosis of Postoperative Complications after Surgery for Hirschsprung Disease**. *Journal of Gastrointestinal Surgery* 2018, **22**(2):335-343.

4. Hui W, Xia-Ne G, Dan Z, Tianqi Z, Lu-Hong H, Jie-Xiong F, Wang H, Guo X-N, Zhu D, Zhu T *et al*: **Nursing Intervention for Outpatient Rehabilitation in Pediatric Patients with Hirschsprung Disease after Colectomy**. *European Journal of Pediatric Surgery* 2015, **25**(05):435-440.

5. Li Q, Li L, Jiang Q, Zhang Z, Xiao P: **The mid-term outcomes of TRM–PIAS, proctocolectomy and ileoanal anastomosis for total colonic aganglionosis**. *Pediatric Surgery International* 2016, **32**(5):477-482.

6. Madbouly KM, Youssef M: **Laparoscopic Ventral Rectopexy Versus Laparoscopic Wells Rectopexy for Complete Rectal Prolapse: Long-Term Results**. *Journal of Laparoendoscopic & Advanced Surgical Techniques* 2018, **28**(1):1-6.

7. Miyano G, Nakamura H, Seo S, Sueyoshi R, Okawada M, Doi T, Koga H, Lane GJ, Okazaki T, Yamataka A: **Rectal mucosal dissection commencing directly on the anorectal line versus commencing above the dentate line in laparoscopy-assisted transanal pull-through for Hirschsprung's disease: Prospective medium-term follow-up**. *Journal of Pediatric Surgery* 2015, **50**(12):2014-2043.

8. Miyano G, Ochi T, Lane GJ, Okazaki T, Yamataka A: **Factors affected by surgical technique when treating total colonic aganglionosis: laparoscopy-assisted versus open surgery**. *Pediatric Surgery International* 2013, **29**(4):349-352.

9. Moore SW: **Genetic impact on the treatment & management of Hirschsprung disease**. *Journal of Pediatric Surgery* 2016, **52**(2):218-222.

10. Moore SW, Tshifularo N: **Hirschsprung’s disease in the neurologically challenged child**. *International journal of adolescent medicine and health* 2011, **23**(3):223-227.

11. More K, Rao S, McMichael J, Minutillo C: **Growth and Developmental Outcomes of Infants with Hirschsprung Disease Presenting in the Neonatal Period: A Retrospective Study**. *The Journal of Pediatrics* 2014, **165**(1):73-77.

12. Nakamura H, Henderson D, Puri P: **A meta-analysis of clinical outcome of intestinal transplantation in patients with total intestinal aganglionosis**. *Pediatric Surgery International* 2017, **33**(8):837-841.

13. Xiaogeng D, Yaohao W, LeXiang Z, Jie Z, Jiajia Z, Ronglin Q, Deng X, Wu Y, Zeng L, Zhang J *et al*: **Comparative Analysis of Modified Laparoscopic Swenson and Laparoscopic Soave Procedure for Short-Segment Hirschsprung Disease in Children**. *European Journal of Pediatric Surgery* 2015, **25**(5):430-434.

14. Xu B, Chuancheng S, Zhongrong W, Yilin S, Hua S, Liang W, Xiyang Y, Bing X, Sun C, Wang Z *et al*: **Transanal pullthrough Soave and Swenson techniques for pediatric patients with Hirschsprung disease**. *Medicine* 2017, **96**(10):e6209.

15. Yokota K, Uchida H, Tainaka T, Tanaka Y, Shirota C, Hinoki A, Kato T, Sumida W, Oshima K, Chiba K *et al*: **Single-stage laparoscopic transanal pull-through modified Swenson procedure without leaving a muscular cuff for short- and long-type Hirschsprung disease: a comparative study**. *Pediatric Surgery International* 2018, **34**(10):1105-1110.

16. Tang ST, Wang GB, Cao GQ, Wang Y, Mao YZ, Li SW, Li S, Yang Y, Yang J, Yang L: **10 years of experience with laparoscopic-assisted endorectal Soave pull-through procedure for Hirschsprung's disease in China**. *J Laparoendosc Adv Surg Tech A* 2012, **22**(3):280-284.

17. Sherman JO, Snyder ME, Weitzman JJ, Jona JZ, Gillis DA, O'Donnell B, Carcassonne M, Swenson O: **A 40-year multinational retrospective study of 880 Swenson procedures**. *J Pediatr Surg* 1989, **24**(8):833-838.

18. Hyman PE: **Adolescents and young adults with Hirschsprung's disease**. *Current gastroenterology reports* 2006, **8**(5):425-429.

19. Granstrom AL, Danielson J, Husberg B, Nordenskjold A, Wester T: **Adult outcomes after surgery for Hirschsprung's disease: Evaluation of bowel function and quality of life**. *J Pediatr Surg* 2015, **50**(11):1865-1869.

20. De la Torre L, Cogley K, Santos K, Morales O, Calisto J: **The anal canal is the fine line between "fecal incontinence and colitis" after a pull-through for Hirschsprung disease**. *J Pediatr Surg* 2017, **52**(12):2011-2017.

21. Stensrud KJ, Emblem R, Bjornland K: **Anal endosonography and bowel function in patients undergoing different types of endorectal pull-through procedures for Hirschsprung disease**. *J Pediatr Surg* 2015, **50**(8):1341-1346.

22. Pratap A, Gupta DK, Shakya VC, Adhikary S, Tiwari A, Shrestha P, Pandey SR, Yadav RK: **Analysis of problems, complications, avoidance and management with transanal pull-through for Hirschsprung disease**. *J Pediatr Surg* 2007, **42**(11):1869-1876.

23. Zaslavsky C, Loening-Baucke V: **Anorectal manometric evaluation of children and adolescents postsurgery for Hirschsprung's disease**. *J Pediatr Surg* 2003, **38**(2):191-195.

24. Witvliet M, Sleeboom C, de Jong J, van Dijk A, Zwaveling S, van der Steeg A: **Anxiety and quality of life of parents with children diagnosed with an anorectal malformation or Hirschsprung disease**. *Eur J Pediatr Surg* 2014, **24**(1):70-74.

25. Chung PHY, Wong KKY, Tam PKH, Leung MWY, Chao NSY, Liu KKW, Chan EKW, Tam YH, Lee KH: **Are all patients with short segment Hirschsprung's disease equal? A retrospective multicenter study**. *Pediatr Surg Int* 2018, **34**(1):47-53.

26. Drissi F, Baayen C, Guinot A, Podevin G, Wyart V, Cretolle C, Lehur PA: **Are Hirschsprung's disease and anorectal malformations sequelae comparable at adult age? Results of disease specific HAQL questionnaire comparison**.

27. Aworanti O, Hung J, McDowell D, Martin I, Quinn F: **Are routine dilatations necessary post pull-through surgery for Hirschsprung disease?** *Eur J Pediatr Surg* 2013, **23**(5):383-388.

28. Zakaria OM: **Bowel function and fecal continence after Soave's trans-anal endorectal pull-through for Hirschsprung's disease: a local experience**. *Updates Surg* 2012, **64**(2):113-118.

29. Jarvi K, Laitakari EM, Koivusalo A, Rintala RJ, Pakarinen MP: **Bowel function and gastrointestinal quality of life among adults operated for Hirschsprung disease during childhood: a population-based study**. *Ann Surg* 2010, **252**(6):977-981.

30. Neuvonen MI, Kyrklund K, Rintala RJ, Pakarinen MP: **Bowel function and quality of life after transanal endorectal pull-through for hirschsprung disease**. *Annals of Surgery* 2017.

31. Diseth TH, Bjornland K, Novik TS, Emblem R: **Bowel function, mental health, and psychosocial function in adolescents with Hirschsprung's disease**. *Arch Dis Child* 1997, **76**(2):100-106.

32. Joosten K, Pruszczynski M, Severijnen RS, Festen C: **Causes of late complications in children operated on for Hirschsprung's disease: a preliminary immunohistochemical investigation using polyclonal antibodies against S-100 protein**. *Zeitschrift fur Kinderchirurgie : organ der Deutschen, der Schweizerischen und der Osterreichischen Gesellschaft fur Kinderchirurgie = Surgery in infancy and childhood* 1989, **44**(4):213-215.

33. Hondel D, Aarsen FK, Wijnen RMH, Sloots CEJ, Ijsselstijn H: **Children with congenital colorectal malformations often require special education or remedial teaching, despite normal intelligence**. 2016.

34. van den Hondel D, Aarsen FK, Wijnen RM, Sloots CE, H IJ: **Children with congenital colorectal malformations often require special education or remedial teaching, despite normal intelligence**. *Acta Paediatr* 2016, **105**(2):e77-84.

35. Dutta HK: **Clinical experience with a new modified transanal endorectal pull-through for Hirschsprung's disease**.

36. Khazdouz M, Sezavar M, Imani B, Akhavan H, Babapour A, Khademi G: **Clinical outcome and bowel function after surgical treatment in Hirschsprung's disease**. *Afr J Paediatr Surg* 2015, **12**(2):143-147.

37. Moore SW, Albertyn R, Cywes S: **Clinical outcome and long-term quality of life after surgical correction of Hirschsprung's disease**. *J Pediatr Surg* 1996, **31**(11):1496-1502.

38. Zhang SC, Bai YZ, Wang W, Wang WL: **Clinical outcome in children after transanal 1-stage endorectal pull-through operation for Hirschsprung disease**. *J Pediatr Surg* 2005, **40**(8):1307-1311.

39. Kubota A, Kawahara H, Okuyama H, Oue T, Tazuke Y, Okada A: **Clinical outcome of laparoscopically assisted endorectal pull-through in Hirschsprung's disease: comparison of abdominal and perineal approaches**. *J Pediatr Surg* 2004, **39**(12):1835-1837.

40. Aubdoollah TH, Li K, Zhang X, Li S, Yang L, Lei HY, Dolo PR, Xiang XC, Cao GQ, Wang GB *et al*: **Clinical outcomes and ergonomics analysis of three laparoscopic techniques for Hirschsprung's disease**. *World J Gastroenterol* 2015, **21**(29):8903-8911.

41. Di Lorenzo C, Solzi GF, Flores AF, Schwankovsky L, Hyman PE: **Colonic motility after surgery for Hirschsprung's disease**. *The American journal of gastroenterology* 2000, **95**(7):1759-1764.

42. Aggarwal SK, Yadav S, Goel D, Sengar M: **Combined abdominal and posterior sagittal approach for redo pull-through operation in Hirschsprung's disease**. *J Pediatr Surg* 2002, **37**(8):1156-1159.

43. Deng X, Wu Y, Zeng L, Zhang J, Zhou J, Qiu R: **Comparative Analysis of Modified Laparoscopic Swenson and Laparoscopic Soave Procedure for Short-Segment Hirschsprung Disease in Children**. *Eur J Pediatr Surg* 2015, **25**(5):430-434.

44. Aworanti OM, McDowell DT, Martin IM, Hung J, Quinn F: **Comparative review of functional outcomes post surgery for Hirschsprung's disease utilizing the paediatric incontinence and constipation scoring system**. *Pediatr Surg Int* 2012, **28**(11):1071-1078.

45. Fujiwara N, Kaneyama K, Okazaki T, Lane GJ, Kato Y, Kobayashi H, Yamataka A: **A comparative study of laparoscopy-assisted pull-through and open pull-through for Hirschsprung's disease with special reference to postoperative fecal continence**. *J Pediatr Surg* 2007, **42**(12):2071-2074.

46. Shinall MC, Jr., Koehler E, Shyr Y, Lovvorn HN, 3rd: **Comparing cost and complications of primary and staged surgical repair of neonatally diagnosed Hirschsprung's disease**. *J Pediatr Surg* 2008, **43**(12):2220-2225.

47. Imvised T, Vejchapipat P, Chiengkriwate P, Thepsuwan P, Tiansri K, Kiatipunsodsai S: **Comparison of Cleaning up Eyelids after Taking off False Eyelashes between Eye Makeup Remover Alone and Eye Makeup Remover with Ocusoft(R)**. *J Med Assoc Thai* 2016, **99 Suppl 4**:S59-64.

48. Minford JL, Ram A, Turnock RR, Lamont GL, Kenny SE, Rintala RJ, Lloyd DA, Baillie CT: **Comparison of functional outcomes of Duhamel and transanal endorectal coloanal anastomosis for Hirschsprung's disease**. *J Pediatr Surg* 2004, **39**(2):161-165; discussion 161-165.

49. Shisong Z, Juan L, Yurui W, Yuanjun H, Chunhong D, Meiyun W, Zhongtao G, Zhang S, Li J, Wu Y *et al*: **Comparison of Laparoscopic-Assisted Operations and Laparotomy Operations for the Treatment of Hirschsprung Disease**. *Medicine* 2015.

50. Shen C, Song Z, Zheng S, Xiao X: **A comparison of the effectiveness of the Soave and Martin procedures for the treatment of total colonic aganglionosis**. *J Pediatr Surg* 2009, **44**(12):2355-2358.

51. Kohno M, Ikawa H, Konuma K, Masuyama H, Fukumoto H, Ogawa E, Oshikiri T, Takahashi S: **Comparison of the postoperative bowel function between transanal endorectal pull-through and transabdominal pull-through for Hirschsprung's disease: a study of the feces excretion function using an RI-defecogram**. *Pediatr Surg Int* 2009, **25**(11):949-954.

52. Sun X, Ren H, Chen S, Wu X, Zhao B, Jin Y, Chen L: **[Complication analysis of endorectal pull-through radical operation for Hirschsprung disease]**. *Zhonghua Wei Chang Wai Ke Za Zhi* 2015, **18**(5):459-462.

53. Tariq GM, Brereton RJ, Wright VM: **Complications of endorectal pull-through for Hirschsprung's disease**. *J Pediatr Surg* 1991, **26**(10):1202-1206.

54. Swenson O: **Congenital megacolon (Hirschsprung's disease); follow-up on eighty-two patients treated surgically**. *Pediatrics* 1951, **8**(4):542-547.

55. Garrett KM, Levitt MA, Pena A, Kraus SJ: **Contrast enema findings in patients presenting with poor functional outcome after primary repair for Hirschsprung disease**. *Pediatr Radiol* 2012, **42**(9):1099-1106.

56. Bischoff A, Frischer J, Knod JL, Dickie B, Levitt MA, Holder M, Jackson L, Pena A: **Damaged anal canal as a cause of fecal incontinence after surgical repair for Hirschsprung disease - a preventable and under-reported complication**. *J Pediatr Surg* 2017, **52**(4):549-553.

57. Teitelbaum DH, Cilley RE, Sherman NJ, Bliss D, Uitvlugt ND, Renaud EJ, Kirstioglu I, Bengston T, Coran AG: **A decade of experience with the primary pull-through for hirschsprung disease in the newborn period: a multicenter analysis of outcomes**. *Ann Surg* 2000, **232**(3):372-380.

58. Chumpitazi BP, Nurko S: **Defecation disorders in children after surgery for Hirschsprung disease**. *J Pediatr Gastroenterol Nutr* 2011, **53**(1):75-79.

59. van Kuyk EM, Brugman-Boezeman AT, Wissink-Essink M, Oerlemans HM, Severijnen RS, Bleijenberg G: **Defecation problems in children with Hirschsprung's disease: a prospective controlled study of a multidisciplinary behavioural treatment**. *Acta Paediatr* 2001, **90**(10):1153-1159.

60. Lane VA, Nacion KM, Cooper JN, Levitt MA, Deans KJ, Minneci PC: **Determinants of quality of life in children with colorectal diseases**. *J Pediatr Surg* 2016, **51**(11):1843-1850.

61. Graneli C, Stenstrom P, Borjesson A, Arnbjornsson E: **Development of Frequency of Stools over Time in Children with Hirschsprung Disease Posttransanal Endorectal One-Stage Pull-through**. *Eur J Pediatr Surg* 2015, **25**(4):359-364.

62. Oh C, Lee S, Lee SK, Seo JM: **Difference of Postoperative Stool Frequency in Hirschsprung Disease According to Anastomosis Level in a Single-Stage, Laparoscopy-Assisted Transanal Endorectal Pull-Through Procedure**. *Medicine* 2016, **95**(14):e3092.

63. Aworanti OM, McDowell DT, Martin IM, Quinn F: **Does Functional Outcome Improve with Time Postsurgery for Hirschsprung Disease?** *Eur J Pediatr Surg* 2016, **26**(2):192-199.

64. Farrugia MK, Alexander N, Clarke S, Nash R, Nicholls EA, Holmes K: **Does transitional zone pull-through in Hirschsprung's disease imply a poor prognosis?** *J Pediatr Surg* 2003, **38**(12):1766-1769.

65. Shogo S, Hiromu M, Hock A, Yuhki K, Chen Y, Lee C, Bo L, Pierro A, Seo S, Miyake H *et al*: **Duhamel and Transanal Endorectal Pull-throughs for Hirschsprung’ Disease: A Systematic Review and Meta-analysis**. 2017.

66. Travassos DV, Bax NM, Van der Zee DC: **Duhamel procedure: a comparative retrospective study between an open and a laparoscopic technique**. *Surg Endosc* 2007, **21**(12):2163-2165.

67. Nah SA, de Coppi P, Kiely EM, Curry JI, Drake DP, Cross K, Spitz L, Eaton S, Pierro A: **Duhamel pull-through for Hirschsprung disease: a comparison of open and laparoscopic techniques**. *J Pediatr Surg* 2012, **47**(2):308-312.

68. van der Zee DC, Bax NM: **Duhamel-Martin procedure for Hirschsprung's disease in neonates and infants: one-stage operation**. *J Pediatr Surg* 1996, **31**(7):901-902.

69. Meinds RJ, Eggink MC, Heineman E, Broens PM: **Dyssynergic defecation may play an important role in postoperative Hirschsprung's disease patients with severe persistent constipation: analysis of a case series**. *J Pediatr Surg* 2014, **49**(10):1488-1492.

70. Conway SJ, Craigie RJ, Cooper LH, Turner K, Turnock RR, Lamont GL, Newton S, Baillie CT, Kenny SE: **Early adult outcome of the Duhamel procedure for left-sided Hirschsprung disease--a prospective serial assessment study**. *J Pediatr Surg* 2007, **42**(8):1429-1432.

71. Hau BD, Quynh TA, Anh VH, Liem NT: **Early and late outcomes of primary laparoscopic endorectal colon pull-through leaving a short rectal seromuscular sleeve for Hirschsprung disease**. *J Laparoendosc Adv Surg Tech A* 2011, **21**(1):81-83.

72. Nguyen TL, Bui DH, Tran AQ, Vu TH: **Early and late outcomes of primary laparoscopic endorectal colon pull-through leaving a short rectal seromuscular sleeve for Hirschsprung disease**. *J Pediatr Surg* 2009, **44**(11):2153-2155.

73. Ademuyiwa AO, Bode CO, Idiodi-Thomas HO, Elebute OA: **Early outcome of open primary pull through versus staged pull through in Hirschsprung's disease: a single centre experience from Nigeria**. *Nig Q J Hosp Med* 2012, **22**(3):164-167.

74. Wester T, Rintala RJ: **Early outcome of transanal endorectal pull-through with a short muscle cuff during the neonatal period**. *J Pediatr Surg* 2004, **39**(2):157-160; discussion 157-160.

75. Lukac M, Antunovic SS, Vujovic D, Petronic I, Nikolic D, Radlovic V, Krstajic T, Krstic Z: **Effectiveness of various surgical methods in treatment of Hirschsprung's disease in children**. *Vojnosanitetski pregled* 2016, **73**(3):246-250.

76. Pena A, Hong A: **Effects of anal invasive treatment and incontinence on mental health and psychosocial functioning of adolescents with Hirshsprung's disease and low anorectal anomalies**. *J Pediatr Surg* 1998, **33**(11):1732.

77. Quan B, Chen Q, Jiang J, Ni L, Tang R, Huang Y, Shi Y, Li N: **[Efficacy and safety of the Jinling procedure in the treatment of adult Hirschsprung disease]**. *Zhonghua Wei Chang Wai Ke Za Zhi* 2016, **19**(7):763-768.

78. So HB, Becker JM, Schwartz DL, Kutin ND: **Eighteen years' experience with neonatal Hirschsprung's disease treated by endorectal pull-through without colostomy**. *J Pediatr Surg* 1998, **33**(5):673-675.

79. Krivchenya DY, Silchenko MI, Soroka VP, Pritula VP, Khursin VN: **Endorectal pull-through for Hirschsprung's disease: 17-year review of results in Ukraine**. *Pediatr Surg Int* 2002, **18**(8):718-722.

80. Kim AC, Langer JC, Pastor AC, Zhang L, Sloots CE, Hamilton NA, Neal MD, Craig BT, Tkach EK, Hackam DJ *et al*: **Endorectal pull-through for Hirschsprung's disease-a multicenter, long-term comparison of results: transanal vs transabdominal approach**. *J Pediatr Surg* 2010, **45**(6):1213-1220.

81. Kwendakwema N, Al-Dulaimi R, Presson AP, Zobell S, Stevens AM, Bucher BT, Barnhart DC, Rollins MD: **Enterocolitis and bowel function in children with Hirschsprung disease and trisomy 21**. *J Pediatr Surg* 2016, **51**(12):2001-2004.

82. Zhang SC, Wang WL, Bai YZ, Wang W: **Evaluation of anorectal function after transanal one-stage endorectal pull through operation in children with Hirschsprung's disease**. *Zhongguo dang dai er ke za zhi = Chinese journal of contemporary pediatrics* 2007, **9**(3):188-192.

83. Nasr A, Langer JC: **Evolution of the technique in the transanal pull-through for Hirschsprung's disease: effect on outcome**. *J Pediatr Surg* 2007, **42**(1):36-39; discussion 39-40.

84. Athanasakos E, Starling J, Ross F, Nunn K, Cass D: **An example of psychological adjustment in chronic illness: Hirschsprung's disease**. *Pediatr Surg Int* 2006, **22**(4):319-325.

85. Gilbert MJ, Mello DF, Lima RAG: **EXPERIÊNCIAS DE MÃES DE FILHOS COM DOENÇA DE HIRSCHSPRUNG: SUBSÍDIOS PARA O CUIDADO DE ENFERMAGEM**. 2009.

86. Hartman EE, Oort FJ, Aronson DC, Hanneman MJ, van Heurn E, de Langen ZJ, Madern GC, Rieu PN, van der Zee DC, Looyaard N *et al*: **Explaining change in quality of life of children and adolescents with anorectal malformations or Hirschsprung disease**. *Pediatrics* 2007, **119**(2):e374-383.

87. Hartman EE, Oort FJ, Visser MR, Sprangers MA, Hanneman MJ, de Langen ZJ, va Heurn LW, Rieu PN, Madern GC, van der Zee DC *et al*: **Explaining change over time in quality of life of adult patients with anorectal malformations or Hirschsprung's disease**. *Diseases of the colon and rectum* 2006, **49**(1):96-103.

88. Lu WT, Chen CC: **Factors affecting postoperative fecal soiling in Hirschsprung's disease**. *Journal of the Formosan Medical Association = Taiwan yi zhi* 1998, **97**(3):170-173.

89. Hartman EE, Oort FJ, Sprangers MA, Hanneman MJ, van Heurn LW, de Langen ZJ, Madern GC, Rieu PN, van der Zee DC, Looyaard N *et al*: **Factors affecting quality of life of children and adolescents with anorectal malformations or Hirschsprung disease**. *J Pediatr Gastroenterol Nutr* 2008, **47**(4):463-471.

90. Tang W, Geng Q, Zhang J, Chen H, Lyu X, Lu C, Jiang W, Li W, Li B, Xu X: **[Fast track surgery combined with laparoscopy in the treatment of infant Hirschsprung disease]**. *Zhonghua Wei Chang Wai Ke Za Zhi* 2014, **17**(8):805-808.

91. Yagi M, Kubota M, Kanada S, Kinoshita Y, Okuyama N, Yamazaki S, Murata H, Hirayama Y: **Fecoflowmetric profiles in postoperative patients with Hirschsprung's disease**. *J Pediatr Surg* 2005, **40**(3):551-554.

92. Mousavi SR, Mehdikhah Z, Kavyani A: **Fish mouth and parachute surgical technique for hirschsprung's disease: our experience in 254 cases with a modified form of Duhamel-Martin procedure**. *Diseases of the colon and rectum* 2008, **51**(10):1559-1561.

93. Swenson O: **Follow up on 200 patients treated for Hirschsprung's disease during a ten-year period**. *Ann Surg* 1957, **146**(4):706-714.

94. Huang Y, Zheng S, Xiao X: **A follow-up study on postoperative function after a transanal Soave 1-stage endorectal pull-through procedure for Hirschsprung's disease**. *J Pediatr Surg* 2008, **43**(9):1691-1695.

95. Archibong AE, Ndoma-Egba R, Umoh MS: **Functional disturbances in children after ano-rectal surgery**. *East African medical journal* 2003, **80**(11):592-594.

96. Stensrud KJ, Emblem R, Bjornland K: **Functional outcome after operation for Hirschsprung disease--transanal vs transabdominal approach**. *J Pediatr Surg* 2010, **45**(8):1640-1644.

97. El-Hak NAG, El-Hemaly MM, Negm EH, El-Hanafy EA, Messeh MHA, Bary HHA: **Functional Outcome after Swenson's Operation for Hirshsprung's Disease**. *Saudi J Gastroenterol* 2010.

98. Gad El-Hak NA, El-Hemaly MM, Negm EH, El-Hanafy EA, Abdel Messeh MH, Abdel Bary HH: **Functional outcome after Swenson's operation for Hirshsprung's disease**. *Saudi journal of gastroenterology : official journal of the Saudi Gastroenterology Association* 2010, **16**(1):30-34.

99. Thakkar HS, Bassett C, Hsu A, Manuele R, Kufeji D, Richards CA, Agrawal M, Keshtgar AS: **Functional outcomes in Hirschsprung disease: A single institution's 12-year experience**. *J Pediatr Surg* 2017, **52**(2):277-280.

100. Marty TL, Seo T, Matlak ME, Sullivan JJ, Black RE, Johnson DG: **Gastrointestinal function after surgical correction of Hirschsprung's disease: long-term follow-up in 135 patients**. *J Pediatr Surg* 1995, **30**(5):655-658.

101. Bazo M, Bailez M: **Health-related quality of life in children and adolescents undergoing surgery for Hirschsprung's disease and anorectal malformations**. *Archivos argentinos de pediatria* 2013, **111**(1):37-44.

102. Wang G, Sun XY, Wei MF, Weng YZ: **Heart-shaped anastomosis for Hirschsprung's disease: Operative technique and long-term follow-up**. *World J Gastroenterol* 2005, **11**(2):296-298.

103. Cram RW: **Hirschprung's disease: long-term follow-up of 65 cases**. *Canadian journal of surgery Journal canadien de chirurgie* 1982, **25**(4):435-437.

104. Levitt MA, Martin CA, Olesevich M, Bauer CL, Jackson LE, Pena A: **Hirschsprung disease and fecal incontinence: diagnostic and management strategies**. *J Pediatr Surg* 2009, **44**(1):271-277; discussion 277.

105. Pini Prato A, Gentilino V, Giunta C, Avanzini S, Mattioli G, Parodi S, Martucciello G, Jasonni V: **Hirschsprung disease: do risk factors of poor surgical outcome exist?** *J Pediatr Surg* 2008, **43**(4):612-619.

106. Miyano G, Takeda M, Koga H, Okawada M, Nakazawa-Tanaka N, Ishii J, Doi T, Lane GJ, Okazaki T, Urao M *et al*: **Hirschsprung’s disease in the laparoscopic transanal pull-through era: implications of age at surgery and technical aspects**. 2017.

107. Zganjer M, Cigit I, Car A, Visnjic S, Butkovic D: **Hirschsprung's disease and Rehbein's procedure--our results in the last 30 years**. *Collegium antropologicum* 2006, **30**(4):905-907.

108. Travassos D, van Herwaarden-Lindeboom M, van der Zee DC: **Hirschsprung's disease in children with Down syndrome: a comparative study**. *Eur J Pediatr Surg* 2011, **21**(4):220-223.

109. Mabula JB, Kayange NM, Manyama M, Chandika AB, Rambau PF, Chalya PL: **Hirschsprung's disease in children: a five year experience at a university teaching hospital in northwestern Tanzania**. *BMC research notes* 2014, **7**:410.

110. Ameh EA, Chirdan LB, Dogo PM, Nmadu PT: **Hirschsprung's disease in the newborn: experience in Zaria, Nigeria**. *Annals of tropical paediatrics* 2001, **21**(4):339-342.

111. Bradnock TJ, Knight M, Kenny S, Nair M, Walker GM: **Hirschsprung's disease in the UK and Ireland: incidence and anomalies**. *Arch Dis Child* 2017, **102**(8):722-727.

112. Sharma S, Gupta DK: **Hirschsprung's disease presenting beyond infancy: surgical options and postoperative outcome**. *Pediatr Surg Int* 2012, **28**(1):5-8.

113. Rescorla FJ, Morrison AM, Engles D, West KW, Grosfeld JL: **Hirschsprung's disease. Evaluation of mortality and long-term function in 260 cases**. *Arch Surg* 1992, **127**(8):934-941; discussion 941-932.

114. Pini Prato A, Gentilino V, Giunta C, Avanzini S, Parodi S, Mattioli G, Martucciello G, Jasonni V: **Hirschsprung's disease: 13 years' experience in 112 patients from a single institution**. *Pediatr Surg Int* 2008, **24**(2):175-182.

115. Reding R, de Ville de Goyet J, Gosseye S, Clapuyt P, Sokal E, Buts JP, Gibbs P, Otte JB: **Hirschsprung's disease: a 20-year experience**. *J Pediatr Surg* 1997, **32**(8):1221-1225.

116. Ludman L, Spitz L, Tsuji H, Pierro A: **Hirschsprung's disease: functional and psychological follow up comparing total colonic and rectosigmoid aganglionosis**. *Arch Dis Child* 2002, **86**(5):348-351.

117. Bandre E, Kabore RA, Ouedraogo I, Sore O, Tapsoba T, Bambara C, Wandaogo A: **Hirschsprung's disease: management problem in a developing country**. *Afr J Paediatr Surg* 2010, **7**(3):166-168.

118. Erdek MA, Wilt E: **Hirschsprung's disease: one institution's ten year experience and long-term follow-up**. *The American surgeon* 1994, **60**(8):625-628.

119. Jung PM: **Hirschsprung's disease: one surgeon's experience in one institution**. *J Pediatr Surg* 1995, **30**(5):646-651.

120. Ghose SI, Squire BR, Stringer MD, Batcup G, Crabbe DC: **Hirschsprung's disease: problems with transition-zone pull-through**. *J Pediatr Surg* 2000, **35**(12):1805-1809.

121. Klein MD, Philippart AI: **Hirschsprung's disease: three decades' experience at a single institution**. *J Pediatr Surg* 1993, **28**(10):1291-1293; discussion 1293-1294.

122. Pini Prato A, Rossi V, Avanzini S, Mattioli G, Disma N, Jasonni V: **Hirschsprung's disease: what about mortality?** *Pediatr Surg Int* 2011, **27**(5):473-478.

123. Aubdoollah TH, Tang ST, Yang L, Li S, Lei HY, Zhang X: **Hybrid Single-Incision Laparoscopic Approaches for Endorectal Pull-Through in Hirschsprung's Disease**. *J Laparoendosc Adv Surg Tech A* 2015, **25**(7):595-598.

124. Morabito A, Lall A, Gull S, Mohee A, Bianchi A: **The impact of Down's syndrome on the immediate and long-term outcomes of children with Hirschsprung's disease**. *Pediatr Surg Int* 2006, **22**(2):179-181.

125. Nakamura H, Lim T, Puri P: **Inflammatory bowel disease in patients with Hirschsprung’s disease: a systematic review and meta-analysis**. 2017.

126. Quinn FM, Surana R, Puri P: **The influence of trisomy 21 on outcome in children with Hirschsprung's disease**. *J Pediatr Surg* 1994, **29**(6):781-783.

127. Shakya VC, Agrawal CS, Adhikary S: **Initial experience with Soave's transabdominal pull-through: an observational study**. *International journal of surgery (London, England)* 2010, **8**(3):225-228.

128. Kobayashi H, Hirakawa H, Surana R, O'Briain DS, Puri P: **Intestinal neuronal dysplasia is a possible cause of persistent bowel symptoms after pull-through operation for Hirschsprung's disease**. *J Pediatr Surg* 1995, **30**(2):253-257; discussion 257-259.

129. Ure BM, Holschneider AM, Schulten D, Meier-Ruge W: **Intestinal transit time in children with intestinal neuronal malformations mimicking Hirschsprung's disease**. *Eur J Pediatr Surg* 1999, **9**(2):91-95.

130. Kohno M, Ikawa H, Konuma K, Masuyama H, Fukumoto H, Morimura E: **Is high amplitude propagated contraction present after transanal endorectal pull-through for Hirschsprung's disease?** *Pediatr Surg Int* 2007, **23**(10):981-986.

131. Tander B, Rizalar R, Cihan AO, Ayyildiz SH, Ariturk E, Bernay F: **Is there a hidden mortality after one-stage transanal endorectal pull-through for patients with Hirschsprung's disease?** *Pediatr Surg Int* 2007, **23**(1):81-86.

132. Thomson D, Allin B, Long AM, Bradnock T, Walker G, Knight M: **Laparoscopic assistance for primary transanal pull-through in Hirschsprung's disease: a systematic review and meta-analysis**. *BMJ open* 2015, **5**(3):e006063.

133. Scholfield DW, Ram AD: **Laparoscopic Duhamel Procedure for Hirschsprung's Disease: Systematic Review and Meta-analysis**. *J Laparoendosc Adv Surg Tech A* 2016, **26**(1):53-61.

134. Kumar R, Mackay A, Borzi P: **Laparoscopic Swenson procedure--an optimal approach for both primary and secondary pull-through for Hirschsprung's disease**. *J Pediatr Surg* 2003, **38**(10):1440-1443.

135. Granström AL, Husberg B, Nordenskjold A, Svensson PJ, Wester T: **Laparoscopic-assisted pull-through for Hirschsprung's disease, a prospective repeated evaluation of functional outcome**. *J Pediatr Surg* 2013, **48**(12):2536-2539.

136. Tomuschat C, Zimmer J, Puri P: **Laparoscopic-assisted pull-through operation for Hirschsprung’s disease: a systematic review and meta-analysis**. *Pediatric Surgery International* 2016, **32**(8):751-757.

137. Guerra J, Wayne C, Musambe T, Nasr A: **Laparoscopic-assisted transanal pull-through (LATP) versus complete transanal pull-through (CTP) in the surgical management of Hirschsprung's disease**. *J Pediatr Surg* 2016, **51**(5):770-774.

138. Miyano G, Nakamura H, Seo S, Sueyoshi R, Okawada M, Doi T, Koga H, Lane GJ, Okazaki T, Yamataka A: **Laparoscopy-Assisted Duhamel-Z Anastomosis for Total Colonic Aganglionosis: Outcome Assessed by Fecal Continence Evaluation**. *J Laparoendosc Adv Surg Tech A* 2017, **27**(3):302-305.

139. Xia X, Li N, Wei J, Zhang W, Yu D, Zhu T, Feng J: **Laparoscopy-assisted versus transabdominal reoperation in Hirschprung's disease for residual aganglionosis and transition zone pathology after transanal pull-through**. *J Pediatr Surg* 2016, **51**(4):577-581.

140. Ghirardo V, Betalli P, Mognato G, Gamba P: **Laparotomic versus laparoscopic Duhamel pull-through for Hirschsprung disease in infants and children**. *J Laparoendosc Adv Surg Tech A* 2007, **17**(1):119-123.

141. Vorm HN, Jensen SI, Qvist N: **Lateral sphincteromyotomy in patients with outlet obstruction after surgery for Hirschsprung's disease and short-segment disease**. *Pediatr Surg Int* 2002, **18**(5-6):368-370.

142. Xiong X, Chen X, Wang G, Feng J: **Long term quality of life in patients with Hirschsprung's disease who underwent heart-shaped anastomosis during childhood: A twenty-year follow-up in China**. *J Pediatr Surg* 2015, **50**(12):2044-2047.

143. Heikkinen M, Rintala R, Luukkonen P: **Long-term anal sphincter performance after surgery for Hirschsprung's disease**. *J Pediatr Surg* 1997, **32**(10):1443-1446.

144. Mills JL, Konkin DE, Milner R, Penner JG, Langer M, Webber EM: **Long-term bowel function and quality of life in children with Hirschsprung's disease**. *J Pediatr Surg* 2008, **43**(5):899-905.

145. Menezes M, Puri P: **Long-term clinical outcome in patients with Hirschsprung's disease and associated Down's syndrome**. *J Pediatr Surg* 2005, **40**(5):810-812.

146. Menezes M, Pini Prato A, Jasonni V, Puri P: **Long-term clinical outcome in patients with total colonic aganglionosis: a 31-year review**. *J Pediatr Surg* 2008, **43**(9):1696-1699.

147. Moore SW, Millar AJ, Cywes S: **Long-term clinical, manometric, and histological evaluation of obstructive symptoms in the postoperative Hirschsprung's patient**. *J Pediatr Surg* 1994, **29**(1):106-111.

148. Catto-Smith AG, Trajanovska M, Taylor RG: **Long-term continence after surgery for Hirschsprung's disease**. *J Gastroenterol Hepatol* 2007, **22**(12):2273-2282.

149. Catto-Smith AG, Trajanovska M, Taylor RG: **Long-term continence in patients with Hirschsprung's disease and Down syndrome**. *J Gastroenterol Hepatol* 2006, **21**(4):748-753.

150. Jiao C, Yu D, Li D, Wang G, Feng J: **A Long-Term Follow-Up of a New Surgery Method: Laparoscope-Assisted Heart-Shaped Anastomosis for Hirschsprung’s Disease**. *JOURNAL OF LAPAROENDOSCOPIC & ADVANCED SURGICAL TECHNIQUES* 2018.

151. Agarwala S, Bhatnagar V, Mitra DK: **Long-term follow-up of Hirschsprung's disease: review of early and late complications**. *Indian Pediatr* 1996, **33**(5):382-386.

152. Emslie J, Krishnamoorthy M, Applebaum H: **Long-term follow-up of patients treated with ileoendorectal pull-through and right colon onlay patch for total colonic aganglionosis**. *J Pediatr Surg* 1997, **32**(11):1542-1544.

153. van Leeuwen K, Teitelbaum DH, Elhalaby EA, Coran AG: **Long-term follow-up of redo pull-through procedures for Hirschsprung's disease: efficacy of the endorectal pull-through**. *J Pediatr Surg* 2000, **35**(6):829-833; discussion 833-824.

154. Liem NT, Hau BD, Thu NX: **The long-term follow-up result of Swenson's operation in the treatment of Hirschsprung's disease in Vietnamese children**. *Eur J Pediatr Surg* 1995, **5**(2):110-112.

155. Bjornland K, Diseth TH, Emblem R: **Long-term functional, manometric, and endosonographic evaluation of patients operated upon with the Duhamel technique**. *Pediatr Surg Int* 1998, **13**(1):24-28.

156. Khalil M: **Long-term health-related quality of life for patients with Hirschsprung's disease at 5 years after transanal endorectal pull-through operation**. *Quality of life research : an international journal of quality of life aspects of treatment, care and rehabilitation* 2015, **24**(11):2733-2738.

157. Hoehner JC, Ein SH, Shandling B, Kim PC: **Long-term morbidity in total colonic aganglionosis**. *J Pediatr Surg* 1998, **33**(7):961-965; discussion 965-966.

158. Yanchar NL, Soucy P: **Long-term outcome after Hirschsprung's disease: patients' perspectives**. *J Pediatr Surg* 1999, **34**(7):1152-1160.

159. Baillie CT, Kenny SE, Rintala RJ, Booth JM, Lloyd DA: **Long-term outcome and colonic motility after the Duhamel procedure for Hirschsprung's disease**. *J Pediatr Surg* 1999, **34**(2):325-329.

160. Bai Y, Chen H, Hao J, Huang Y, Wang W: **Long-term outcome and quality of life after the Swenson procedure for Hirschsprung's disease**. *J Pediatr Surg* 2002, **37**(4):639-642.

161. Onishi S, Nakame K, Yamada K, Yamada W, Kawano T, Mukai M, Kaji T, Ieiri S: **Long-term outcome of bowel function for 110 consecutive cases of Hirschsprung's disease: Comparison of the abdominal approach with transanal approach more than 30years in a single institution - is the transanal approach truly beneficial for bowel function?** *J Pediatr Surg* 2016, **51**(12):2010-2014.

162. Dahal GR, Wang JX, Guo LH: **Long-term outcome of children after single-stage transanal endorectal pull-through for Hirschsprung's disease**. *World journal of pediatrics : WJP* 2011, **7**(1):65-69.

163. Muller CO, Rossignol G, Montalva L, Viala J, Martinez-Vinson C, Mosca A, Berrebi D, Bonnard A: **Long-Term Outcome of Laparoscopic Duhamel Procedure for Extended Hirschsprung's Disease**. *J Laparoendosc Adv Surg Tech A* 2016, **26**(12):1032-1035.

164. Raboei EH: **Long-term outcome of total colonic aganglionosis**. *Eur J Pediatr Surg* 2008, **18**(5):300-302.

165. Zhang SC, Bai YZ, Wang W, Wang WL: **Long-term outcome, colonic motility, and sphincter performance after Swenson's procedure for Hirschsprung's disease: a single-center 2-decade experience with 346 cases**. *Am J Surg* 2007, **194**(1):40-47.

166. Wang L, He Q, Jiang J, Li N: **Long-term outcomes and quality of life after subtotal colectomy combined with modified Duhamel procedure for adult Hirschsprung's disease**. *Pediatr Surg Int* 2014, **30**(1):55-61.

167. Suita S, Taguchi T, Yanai K, Kamimura T, Nakao M, Ikeda K: **Longterm outcomes and quality of life after Z-shaped anastomosis for Hirschsprung's disease**. *J Am Coll Surg* 1998, **187**(6):577-583.

168. Ieiri S, Nakatsuji T, Akiyoshi J, Higashi M, Hashizume M, Suita S, Taguchi T: **Long-term outcomes and the quality of life of Hirschsprung disease in adolescents who have reached 18 years or older--a 47-year single-institute experience**. *J Pediatr Surg* 2010, **45**(12):2398-2402.

169. Escobar MA, Grosfeld JL, West KW, Scherer LR, Rouse TM, Engum SA, Rescorla FJ: **Long-term outcomes in total colonic aganglionosis: a 32-year experience**. *J Pediatr Surg* 2005, **40**(6):955-961.

170. Erginel B, Gun Soysal F, Keskin E, Celik A, Yuksel S, Salman T: **Long-term outcomes of children with transanal endorectal pull-through and a review of the literature**. *Acta Chir Belg* 2016, **116**(6):372-375.

171. Rintala RJ, Pakarinen MP: **Long-term outcomes of Hirschsprung's disease**. *Semin Pediatr Surg* 2012, **21**(4):336-343.

172. Menezes M, Corbally M, Puri P: **Long-term results of bowel function after treatment for Hirschsprung's disease: a 29-year review**. *Pediatr Surg Int* 2006, **22**(12):987-990.

173. Rassouli R, Holschneider AM, Bolkenius M, Menardi G, Becker MR, Schaarschmidt K, Illing P, Hagel CI, Holland-Cunz S, Loffler W *et al*: **Long-term results of Rehbein's procedure: a retrospective study in German-speaking countries**. *Eur J Pediatr Surg* 2003, **13**(3):187-194.

174. Davenport M: **Long-term results of surgical treatment in infants with total colonic aganglionosis**. *J Pediatr Surg* 1995, **30**(6):901.

175. Endo M, Watanabe K, Fuchimoto Y, Ikawa H, Yokoyama J: **Long-term results of surgical treatment in infants with total colonic aganglionosis**. *J Pediatr Surg* 1994, **29**(10):1310-1314.

176. Tannuri AC, Ferreira MA, Mathias AL, Tannuri U: **Long-term results of the Duhamel technique are superior to those of the transanal pullthrough: A study of fecal continence and quality of life**. *J Pediatr Surg* 2017, **52**(3):449-453.

177. Barrena S, Andres AM, Burgos L, Luis AL, Hernandez F, Martinez L, Lopez-Santamaria M, Lassaletta L, Tovar JA: **Long-term results of the treatment of total colonic aganglionosis with two different techniques**. *Eur J Pediatr Surg* 2008, **18**(6):375-379.

178. Amerstorfer EE, Fasching G, Till H, Huber-Zeyringer A, Hollwarth ME: **Long-term results of total colonic agangliosis patients treated by preservation of the aganglionic right hemicolon and the ileo-cecal valve**. *Pediatr Surg Int* 2015, **31**(8):773-780.

179. Zimmer J, Tomuschat C, Puri P: **Long-term results of transanal pull-through for Hirschsprung's disease: a meta-analysis**. *Pediatr Surg Int* 2016, **32**(8):743-749.

180. Engum SA, Grosfeld JL: **Long-term results of treatment of Hirschsprung's disease**. *Semin Pediatr Surg* 2004, **13**(4):273-285.

181. Teitelbaum DH, Drongowski RA, Chamberlain JN, Coran AG: **Long-term stooling patterns in infants undergoing primary endorectal pull-through for Hirschsprung's disease**. *J Pediatr Surg* 1997, **32**(7):1049-1052; discussion 1052-1043.

182. Neuvonen M, Kyrklund K, Taskinen S, Koivusalo A, Rintala RJ, Pakarinen MP: **Lower urinary tract symptoms and sexual functions after endorectal pull-through for Hirschsprung disease: controlled long-term outcomes**.

183. Tsuji H, Spitz L, Kiely EM, Drake DP, Pierro A: **Management and long-term follow-up of infants with total colonic aganglionosis**. *J Pediatr Surg* 1999, **34**(1):158-161; discussion 162.

184. Ramesh JC, Ramanujam TM, Yik YI, Goh DW: **Management of Hirschsprung's disease with reference to one-stage pull-through without colostomy**. *J Pediatr Surg* 1999, **34**(11):1691-1694.

185. Saleh W, Rasheed K, Mohaidly MA, Kfoury H, Tariq M, Rawaf AA: **Management of Hirschsprung's disease: a comparison of Soave's and Duhamel's pull-through methods**. *Pediatr Surg Int* 2004, **20**(8):590-593.

186. Sarioglu A, Senocak M, Hicsonmez A, Buyukpamukcu N: **Management of uncommon complications of definitive operations of Hirschsprung's disease**. *Eur J Pediatr Surg* 1996, **6**(6):358-361.

187. Ruttenstock E, Puri P: **A meta-analysis of clinical outcome in patients with total intestinal aganglionosis**. *Pediatr Surg Int* 2009, **25**(10):833-839.

188. Li Q, Li L, Jiang Q, Zhang Z, Xiao P: **The mid-term outcomes of TRM-PIAS, proctocolectomy and ileoanal anastomosis for total colonic aganglionosis**. *Pediatr Surg Int* 2016, **32**(5):477-482.

189. Liem NT, Hau BD, Son HT: **Modified Soave procedure through the posterior sagittal approach for Hirschsprung's disease**. *J Pediatr Surg* 2005, **40**(3):547-550.

190. Miyano T, Yamataka A, Urao M, Kobayashi H, Lane GJ: **Modified soave pull-through for Hirschsprung's disease: intraoperative internal sphincterotomy**. *J Pediatr Surg* 1999, **34**(11):1599-1602.

191. Levy M, Reynolds M: **Morbidity associated with total colon Hirschsprung's disease**. *J Pediatr Surg* 1992, **27**(3):364-366; discussion 367.

192. Lof Granstrom A, Wester T: **Mortality in Swedish patients with Hirschsprung disease**. *Pediatr Surg Int* 2017, **33**(11):1177-1181.

193. Allin BSR, Bradnock T, Kenny S, Kurinczuk JJ, Walker G, Knight M: **NETS(1HD) study: development of a Hirschsprung's disease core outcome set**. *Arch Dis Child* 2017, **102**(12):1143-1151.

194. Li AW, Zhang WT, Li FH, Cui XH, Duan XS: **A new modification of transanal Soave pull-through procedure for Hirschsprung's disease**. *Chin Med J (Engl)* 2006, **119**(1):37-42.

195. Xu ZL, Zhao Z, Wang L, An Q, Tao WF: **A new modification of transanal Swenson pull-through procedure for Hirschsprung's disease**. *Chin Med J (Engl)* 2008, **121**(23):2420-2423.

196. Bjornland K, Pakarinen MP, Stenstrom P, Stensrud KJ, Neuvonen M, Granstrom AL, Graneli C, Pripp AH, Arnbjornsson E, Emblem R *et al*: **A Nordic multicenter survey of long-term bowel function after transanal endorectal pull-through in 200 patients with rectosigmoid Hirschsprung disease**. *J Pediatr Surg* 2017, **52**(9):1458-1464.

197. Wang H, Guo XN, Zhu D, Zhu T, Hu LH, Feng JX: **Nursing Intervention for Outpatient Rehabilitation in Pediatric Patients with Hirschsprung Disease after Colectomy**. *Eur J Pediatr Surg* 2015, **25**(5):435-440.

198. Haricharan RN, Seo JM, Kelly DR, Mroczek-Musulman EC, Aprahamian CJ, Morgan TL, Georgeson KE, Harmon CM, Saito JM, Barnhart DC: **Older age at diagnosis of Hirschsprung disease decreases risk of postoperative enterocolitis, but resection of additional ganglionated bowel does not**. *J Pediatr Surg* 2008, **43**(6):1115-1123.

199. Wilcox DT, Bruce J, Bowen J, Bianchi A: **One-stage neonatal pull-through to treat Hirschsprung's disease**. *J Pediatr Surg* 1997, **32**(2):243-245; discussion 245-247.

200. Liem NT, Hau BD: **One-stage operation for Hirschsprung's disease: experience with 192 cases**. *Asian J Surg* 2008, **31**(4):216-219.

201. Ammar SA, Ibrahim IA: **One-stage transanal endorectal pull-through for treatment of hirschsprung's disease in adolescents and adults**. *Journal of gastrointestinal surgery : official journal of the Society for Surgery of the Alimentary Tract* 2011, **15**(12):2246-2250.

202. Langer JC, Durrant AC, de la Torre L, Teitelbaum DH, Minkes RK, Caty MG, Wildhaber BE, Ortega SJ, Hirose S, Albanese CT: **One-stage transanal Soave pullthrough for Hirschsprung disease: a multicenter experience with 141 children**. *Ann Surg* 2003, **238**(4):569-583; discussion 583-565.

203. Langer JC, Fitzgerald PG, Winthrop AL, Srinathan SK, Foglia RP, Skinner MA, Ternberg JL, Lau GY: **One-stage versus two-stage Soave pull-through for Hirschsprung's disease in the first year of life**. *J Pediatr Surg* 1996, **31**(1):33-36; discussion 36-37.

204. Gosemann JH, Friedmacher F, Ure B, Lacher M: **Open versus transanal pull-through for Hirschsprung disease: a systematic review of long-term outcome**. *Eur J Pediatr Surg* 2013, **23**(2):94-102.

205. Aslan MK, Karaman I, Karaman A, Erdogan D, Cavusoglu YH, Cakmak O: **Our experience with transanal endorectal pull-through in Hirschsprung's disease**. *Eur J Pediatr Surg* 2007, **17**(5):335-339.

206. Giuliani S, Betalli P, Narciso A, Grandi F, Midrio P, Mognato G, Gamba P: **Outcome comparison among laparoscopic Duhamel, laparotomic Duhamel, and transanal endorectal pull-through: a single-center, 18-year experience**. *J Laparoendosc Adv Surg Tech A* 2011, **21**(9):859-863.

207. Rintala RJ, Pakarinen MP: **Outcome of anorectal malformations and Hirschsprung's disease beyond childhood**. *Semin Pediatr Surg* 2010, **19**(2):160-167.

208. Urushihara N, Fukumoto K, Fukuzawa H, Sugiyama A, Watanabe K, Mitsunaga M, Miyake H, Kusafuka J, Aoba T: **Outcome of laparoscopic modified Duhamel procedure with Z-shaped anastomosis for Hirschsprung's disease**. *Surg Endosc* 2012, **26**(5):1325-1331.

209. Mattioli G, Pini Prato A, Giunta C, Avanzini S, Della Rocca M, Montobbio G, Parodi S, Rapuzzi G, Georgeson K, Jasonni V: **Outcome of primary endorectal pull-through for the treatment of classic Hirschsprung disease**. *J Laparoendosc Adv Surg Tech A* 2008, **18**(6):869-874.

210. Shahjahan M, Ferdous MN, Nag UK, Ullah MS, Rahman MA, Islam MK: **Outcome of single-stage transanal endorectal pull through for short segment Hirschsprung's disease in neonates and infants**. *Mymensingh medical journal : MMJ* 2014, **23**(1):69-74.

211. Obermayr F, Szavay P, Beschorner R, Fuchs J: **Outcome of transanal endorectal pull-through in patients with Hirschsprung's disease**. *Eur J Pediatr Surg* 2009, **19**(4):220-223.

212. Romero P, Kroiss M, Chmelnik M, Konigs I, Wessel LM, Holland-Cunz S: **Outcome of transanal endorectal vs. transabdominal pull-through in patients with Hirschsprung's disease**. *Langenbecks Arch Surg* 2011, **396**(7):1027-1033.

213. Osifo OD, Okolo CJ: **Outcome of trans-anal posterior anorectal myectomy for the ultrashort segment Hirschsprung's disease--Benin City experience in five years**. *The Nigerian postgraduate medical journal* 2009, **16**(3):213-217.

214. Huang B, Li WM, Feng ZY, Huang LY: **[Outcomes and defecation after one-stage transanal endorectal pull-through procedure for Hirschsprung disease]**. *Zhonghua Wei Chang Wai Ke Za Zhi* 2012, **15**(7):715-718.

215. Fraser JD, Garey CL, Laituri CA, Sharp RJ, Ostlie DJ, St Peter SD: **Outcomes of laparoscopic and open total colectomy in the pediatric population**. *J Laparoendosc Adv Surg Tech A* 2010, **20**(7):659-660.

216. Choe EK, Moon SB, Kim HY, Lee SC, Park KW, Jung SE: **Outcomes of surgical management of total colonic aganglionosis**. *World J Surg* 2008, **32**(1):62-68.

217. Stenstrom P, Brautigam M, Borg H, Graneli C, Lilja HE, Wester T: **Patient-reported Swedish nationwide outcomes of children and adolescents with total colonic aganglionosis**. *J Pediatr Surg* 2017, **52**(8):1302-1307.

218. Wu X, Feng J, Wei M, Guo X, Li R, Xuan X, Yang J: **Patterns of postoperative enterocolitis in children with Hirschsprung's disease combined with hypoganglionosis**. *J Pediatr Surg* 2009, **44**(7):1401-1404.

219. Albanese CT, Jennings RW, Smith B, Bratton B, Harrison MR: **Perineal one-stage pull-through for Hirschsprung's disease**. *J Pediatr Surg* 1999, **34**(3):377-380.

220. Miele E, Tozzi A, Staiano A, Toraldo C, Esposito C, Clouse RE: **Persistence of abnormal gastrointestinal motility after operation for Hirschsprung's disease**. *The American journal of gastroenterology* 2000, **95**(5):1226-1230.

221. Langer JC: **Persistent obstructive symptoms after surgery for Hirschsprung's disease: development of a diagnostic and therapeutic algorithm**. *J Pediatr Surg* 2004, **39**(10):1458-1462.

222. Granstrom AL, Svenningsson A, Nordenskjold A, Wester T: **Population-based study shows that Hirschsprung disease does not have a negative impact on education and income**. *Acta Paediatr* 2016, **105**(12):1508-1512.

223. Neuvonen MI, Kyrklund K, Lindahl HG, Koivusalo AI, Rintala RJ, Pakarinen MP: **A population-based, complete follow-up of 146 consecutive patients after transanal mucosectomy for Hirschsprung disease**. *J Pediatr Surg* 2015, **50**(10):1653-1658.

224. Wildhaber BE, Pakarinen M, Rintala RJ, Coran AG, Teitelbaum DH: **Posterior myotomy/myectomy for persistent stooling problems in Hirschsprung's disease**. *J Pediatr Surg* 2004, **39**(6):920-926; discussion 920-926.

225. Mishalany HG, Woolley MM: **Postoperative functional and manometric evaluation of patients with Hirschsprung's disease**. *J Pediatr Surg* 1987, **22**(5):443-446.

226. Wester T, Zetterlind L, Fredin K, Olsen L: **Postoperative obstructive symptoms are common after Rehbein's procedure for Hirschsprung's disease**. *Eur J Pediatr Surg* 2006, **16**(2):100-103.

227. Huang SF, Chen CC, Lai HS: **Prediction of the outcome of pull-through surgery for Hirschsprung's disease using acetylcholinesterase activity**. *Journal of the Formosan Medical Association = Taiwan yi zhi* 2001, **100**(12):798-804.

228. Yokoi A, Satoh S, Takamizawa S, Muraji T, Tsugawa C, Nishijima E: **The preliminary study of modified Swenson procedure in Hirschsprung disease**. *J Pediatr Surg* 2009, **44**(8):1560-1563.

229. Murthi GV, Raine PA: **Preoperative enterocolitis is associated with poorer long-term bowel function after Soave-Boley endorectal pull-through for Hirschsprung's disease**. *J Pediatr Surg* 2003, **38**(1):69-72; discussion 69-72.

230. Liem NT, Hau BD: **Primary laparoscopic endorectal colon pull-through for Hirschsprung's disease: early results of 61 cases**. *Asian J Surg* 2006, **29**(3):173-175.

231. Craigie RJ, Conway SJ, Cooper L, Turnock RR, Lamont GL, Baillie CT, Kenny SE: **Primary pull-through for Hirschsprung's disease: comparison of open and laparoscopic-assisted procedures**. *J Laparoendosc Adv Surg Tech A* 2007, **17**(6):809-812.

232. Chiengkriwate P, Patrapinyokul S, Sangkhathat S, Chowchuvech V: **Primary pull-through with modified Duhamel technique: 1 institution's experience**. *J Pediatr Surg* 2007, **42**(6):1075-1080.

233. Santos MC, Giacomantonio JM, Lau HY: **Primary Swenson pull-through compared with multiple-stage pull-through in the neonate**. *J Pediatr Surg* 1999, **34**(7):1079-1081.

234. Sookpotarom P, Vejchapipat P: **Primary transanal Swenson pull-through operation for Hirschsprung's disease**. *Pediatr Surg Int* 2009, **25**(9):767-773.

235. Somme S, Langer JC: **Primary versus staged pull-through for the treatment of Hirschsprung disease**. *Semin Pediatr Surg* 2004, **13**(4):249-255.

236. El-Sawaf M, Siddiqui S, Mahmoud M, Drongowski R, Teitelbaum DH: **Probiotic prophylaxis after pullthrough for Hirschsprung disease to reduce incidence of enterocolitis: a prospective, randomized, double-blind, placebo-controlled, multicenter trial**. 2013.

237. Dickie BH, Webb KM, Eradi B, Levitt MA: **The problematic Soave cuff in Hirschsprung disease: manifestations and treatment**. *J Pediatr Surg* 2014, **49**(1):77-80; discussion 80-71.

238. Yang L, Tang ST: **A prosective study of laparoscopic transanal endorectal pull-through for subtotal colectomy in hirschsprung's disease: anastomosis using long cuff or short cuff?**

239. Ishihara M, Yamataka A, Kaneyama K, Koga H, Kobayashi H, Lane GJ, Miyano T: **Prospective analysis of primary modified Georgeson's laparoscopy-assisted endorectal pull-through for Hirschsprung's disease: short- to mid-term results**. *Pediatr Surg Int* 2005, **21**(11):878-882.

240. Schulten D, Holschneider AM, Meier-Ruge W: **Proximal segment histology of resected bowel in Hirschsprung's disease predicts postoperative bowel function**. *Eur J Pediatr Surg* 2000, **10**(6):378-381.

241. van den Hondel D, Sloots CE, Bolt JM, Wijnen RM, de Blaauw I, H IJ: **Psychosexual Well-Being after Childhood Surgery for Anorectal Malformation or Hirschsprung's Disease**. *The journal of sexual medicine* 2015, **12**(7):1616-1625.

242. Witvliet MJ, Bakx R, Zwaveling S, van Dijk TH, van der Steeg AF: **Quality of Life and Anxiety in Parents of Children with an Anorectal Malformation or Hirschsprung Disease: The First Year after Diagnosis**. *Eur J Pediatr Surg* 2016, **26**(1):2-6.

243. Hartman EE, Oort FJ, Aronson DC, Sprangers MAG: **Quality of life and disease-specifi c functioning of patients with anorectal malformations or Hirschsprung’s disease: a review**. 2010.

244. Hartman EE, Oort FJ, Aronson DC, Sprangers MA: **Quality of life and disease-specific functioning of patients with anorectal malformations or Hirschsprung's disease: a review**. *Arch Dis Child* 2011, **96**(4):398-406.

245. Gunnarsdottir A, Sandblom G, Arnbjornsson E, Larsson LT: **Quality of life in adults operated on for Hirschsprung disease in childhood**. *J Pediatr Gastroenterol Nutr* 2010, **51**(2):160-166.

246. Niramis R, Watanatittan S, Anuntkosol M, Buranakijcharoen V, Rattanasuwan T, Tongsin A, Petlek W, Mahatharadol V: **Quality of Life of Patients with Hirschsprung’s Disease at 5 - 20 Years Post Pull-Through Operations**. *European Journal of Pediatric Surgery* 2008.

247. Collins L, Collis B, Trajanovska M, Khanal R, Hutson JM, Teague WJ, King SK: **Quality of life outcomes in children with Hirschsprung disease**. *J Pediatr Surg* 2017, **52**(12):2006-2010.

248. Marty TL, Seo T, Sullivan JJ, Matlak ME, Black RE, Johnson DG: **Rectal irrigations for the prevention of postoperative enterocolitis in Hirschsprung's disease**. *J Pediatr Surg* 1995, **30**(5):652-654.

249. Ralls MW, Freeman JJ, Rabah R, Coran AG, Ehrlich PF, Hirschl RB, Teitelbaum DH: **Redo pullthrough for Hirschsprung disease: a single surgical group's experience**. *J Pediatr Surg* 2014, **49**(9):1394-1399.

250. Dingemans A, van der Steeg H, Rassouli-Kirchmeier R, Linssen MW, van Rooij I, de Blaauw I: **Redo pull-through surgery in Hirschsprung disease: Short-term clinical outcome**. *J Pediatr Surg* 2017, **52**(9):1446-1450.

251. Pini-Prato A, Mattioli G, Giunta C, Avanzini S, Magillo P, Bisio GM, Jasonni V: **Redo surgery in Hirschsprung disease: what did we learn? Unicentric experience on 70 patients**. *J Pediatr Surg* 2010, **45**(4):747-754.

252. Peng C, Chen Y, Zhang T, Pang W, Wang Z, Wu D: **[Redo surgery in Hirschsprung's disease for postoperative distension and constipation]**. *Zhonghua Wei Chang Wai Ke Za Zhi* 2015, **18**(12):1235-1239.

253. Wester T, Hoehner J, Olsen L: **Rehbein's anterior resection in Hirschsprung's disease, using a circular stapler**. *Eur J Pediatr Surg* 1995, **5**(6):358-362.

254. Fuchs O, Booss D: **Rehbein's procedure for Hirschsprung's disease. An appraisal of 45 years**. *Eur J Pediatr Surg* 1999, **9**(6):389-391.

255. Thepcharoennirund S: **Rehbein's procedure in 73 cases of Hirschsprung's disease**. *J Med Assoc Thai* 2004, **87**(10):1188-1192.

256. Weber TR, Fortuna RS, Silen ML, Dillon PA: **Reoperation for Hirschsprung's disease**. *J Pediatr Surg* 1999, **34**(1):153-156; discussion 156-157.

257. Sheng Q, Lv Z, Xiao X: **Re-operation for Hirschsprung's disease: experience in 24 patients from China**. *Pediatr Surg Int* 2012, **28**(5):501-506.

258. Pena A, Elicevik M, Levitt MA: **Reoperations in Hirschsprung disease**. *J Pediatr Surg* 2007, **42**(6):1008-1013; discussion 1013-1004.

259. Wilcox DT, Kiely EM: **Repeat pull-through for Hirschsprung's disease**. *J Pediatr Surg* 1998, **33**(10):1507-1509.

260. Friedmacher F, Puri P: **Residual aganglionosis after pull-through operation for Hirschsprung's disease: a systematic review and meta-analysis**. *Pediatr Surg Int* 2011, **27**(10):1053-1057.

261. Hukkinen M, Koivusalo A, Rintala RJ, Pakarinen MP: **Restorative proctocolectomy with J-pouch ileoanal anastomosis for total colonic aganglionosis among neonates and infants**. *J Pediatr Surg* 2014, **49**(4):570-574.

262. Mattioli G, Castagnetti M, Martucciello G, Jasonni V: **Results of a mechanical Duhamel pull-through for the treatment of Hirschsprung's disease and intestinal neuronal dysplasia**. *J Pediatr Surg* 2004, **39**(9):1349-1355.

263. Martinez-Criado Y, Cabrera R, Moya MJ, Valladares JC, Lopez-Alonso M, De Agustin Asensio JC: **Results of transanal endorrectal descent in Hirschprung's disease**. *Cirugia espanola* 2015, **93**(9):561-566.

264. Antao B, Radhwan T, Samuel M, Kiely E: **Short-pouch and low-anastomosis Duhamel procedure results in better fecal control and normal defecation pattern**. *Diseases of the colon and rectum* 2005, **48**(9):1791-1796.

265. Tang ST, Yang Y, Li SW, Cao GQ, Yang L, Huang X, Shuai L, Wang GB: **Single-incision laparoscopic versus conventional laparoscopic endorectal pull-through for Hirschsprung's disease: a comparison of short-term surgical results**.

266. Xia X, Li N, Wei J, Zhang W, Yu D, Zhu T, Feng J: **Single-incision laparoscopic versus conventional laparoscopic surgery for Hirschsprung's disease: A comparison of medium-term outcomes**. *J Pediatr Surg* 2016, **51**(3):440-443.

267. Hackam DJ, Superina RA, Pearl RH: **Single-stage repair of Hirschsprung's disease: a comparison of 109 patients over 5 years**. *J Pediatr Surg* 1997, **32**(7):1028-1031; discussion 1031-1022.

268. Lu C, Hou G, Liu C, Geng Q, Xu X, Zhang J, Chen H, Tang W: **Single-stage transanal endorectal pull-through procedure for correction of Hirschsprung disease in neonates and nonneonates: A multicenter study**. *J Pediatr Surg* 2017, **52**(7):1102-1107.

269. Sulkowski JP, Cooper JN, Congeni A, Pearson EG, Nwomeh BC, Doolin EJ, Blakely ML, Minneci PC, Deans KJ: **Single-stage versus multi-stage pull-through for Hirschsprung's disease: practice trends and outcomes in infants**. *J Pediatr Surg* 2014, **49**(11):1619-1625.

270. Krois W, Dingemans AJM, Hernandez PX, Metzelder ML, Craniotis Rios J, Reck-Burneo CA: **Sociodemographics and the impact of a colostomy to indigent families and children with colorectal disorders in Honduras**. *J Pediatr Surg* 2018, **53**(4):841-846.

271. Lawal TA, Olulana DI, Ogundoyin OO: **Spectrum of colorectal surgery operations performed in a single paediatric surgery unit in sub-Saharan Africa**. *Afr J Paediatr Surg* 2014, **11**(2):128-131.

272. Kim HY, Oh JT: **Stabilization period after 1-stage transanal endorectal pull-through operation for Hirschsprung disease**. *J Pediatr Surg* 2009, **44**(9):1799-1804.

273. Van Leeuwen K, Geiger JD, Barnett JL, Coran AG, Teitelbaum DH: **Stooling and manometric findings after primary pull-throughs in Hirschsprung's disease: Perineal versus abdominal approaches**. *J Pediatr Surg* 2002, **37**(9):1321-1325.

274. Zhang SC, Bai YZ, Wang W, Wang WL: **Stooling patterns and colonic motility after transanal one-stage pull-through operation for Hirschsprung's disease in children**. *J Pediatr Surg* 2005, **40**(11):1766-1772.

275. Zhu T, Feng J, Zhang W, Wei M, Yu D, Zhang X, Yu K, Kuang H: **Subtotal colectomy with a single-incision laparoscopic surgery technique in children with long-segment Hirschsprung disease and allied disorders**. *Pediatr Surg Int* 2013, **29**(2):197-201.

276. Nouira F, Ben Ahmed Y, Sarrai N, Ghorbel S, Jlidi S, Khemakhem R, Charieg A, Chaouachi B: **Surgical management of recto-sigmoid Hirschsprung's disease**. *Acta Chir Belg* 2012, **112**(2):126-130.

277. Ademuyiwa AO, Bode CO, Lawal OA, Seyi-Olajide J: **Swenson's pull-through in older children and adults: peculiar peri-operative challenges of surgery**. *International journal of surgery (London, England)* 2011, **9**(8):652-654.

278. Doodnath R, Puri P: **A systematic review and meta-analysis of Hirschsprung's disease presenting after childhood**. *Pediatr Surg Int* 2010, **26**(11):1107-1110.

279. Anupama B, Zheng S, Xiao X: **Ten-year experience in the management of total colonic aganglionosis**. *J Pediatr Surg* 2007, **42**(10):1671-1676.

280. Sowande OA, Adejuyigbe O: **Ten-year experience with the Swenson procedure in Nigerian children with Hirschsprung's disease**. *Afr J Paediatr Surg* 2011, **8**(1):44-48.

281. Xiao S, Yang W, Yuan L, Zhang Y, Song T, Xu L, Tian S, Ge W, Zhou J, Zhu X: **Timing investigation of single-stage definitive surgery for newborn with Hirschsprung's disease**.

282. Dodero P, Magillo P, Scarsi PL: **Total colectomy and straight ileo-anal soave endorectal pull-through: personal experience with 42 cases**. *Eur J Pediatr Surg* 2001, **11**(5):319-323.

283. Laughlin DM, Friedmacher F, Puri P: **Total colonic aganglionosis: a systematic review and meta-analysis of long-term clinical outcome**. *Pediatr Surg Int* 2012, **28**(8):773-779.

284. Wildhaber BE, Teitelbaum DH, Coran AG: **Total colonic Hirschsprung's disease: a 28-year experience**. *J Pediatr Surg* 2005, **40**(1):203-206; discussion 206-207.

285. Sapin E, Centonze A, Moog R, Borgnon J, Becmeur F: **Transanal coloanal anastomosis for Hirschsprung's disease: comparison between endorectal and perirectal pull-through procedures**. *Eur J Pediatr Surg* 2006, **16**(5):312-317.

286. Rintala RJ: **Transanal coloanal pull-through with a short muscular cuff for classic Hirschsprung's disease**. *Eur J Pediatr Surg* 2003, **13**(3):181-186.

287. Shankar KR, Losty PD, Lamont GL, Turnock RR, Jones MO, Lloyd DA, Lindahl H, Rintala RJ: **Transanal endorectal coloanal surgery for Hirschsprung's disease: experience in two centers**. *J Pediatr Surg* 2000, **35**(8):1209-1213.

288. van de Ven TJ, Sloots CE, Wijnen MH, Rassouli R, van Rooij I, Wijnen RM, de Blaauw I: **Transanal endorectal pull-through for classic segment Hirschsprung's disease: with or without laparoscopic mobilization of the rectosigmoid?** *J Pediatr Surg* 2013, **48**(9):1914-1918.

289. Yang L, Tang ST, Cao GQ, Yang Y, Li S, Li SW, Wang Y, Mao YZ, Ruan QL, Wang GB: **Transanal endorectal pull-through for Hirschsprung's disease using long cuff dissection and short V-shaped partially resected cuff anastomosis: early and late outcomes**. *Pediatr Surg Int* 2012, **28**(5):515-521.

290. Adiguzel U, Agengin K, Kiristioglu I, Dogruyol H: **Transanal endorectal pull-through for Hirschsprung's disease: experience with 50 patients**. *Irish journal of medical science* 2017, **186**(2):433-437.

291. Hadidi A: **Transanal endorectal pull-through for Hirschsprung's disease: experience with 68 patients**. *J Pediatr Surg* 2003, **38**(9):1337-1340.

292. Tannuri AC, Tannuri U, Romao RL: **Transanal endorectal pull-through in children with Hirschsprung's disease--technical refinements and comparison of results with the Duhamel procedure**. *J Pediatr Surg* 2009, **44**(4):767-772.

293. Gunnarsdottir A, Larsson LT, Arnbjornsson E: **Transanal endorectal vs. Duhamel pull-through for Hirschsprung's disease**. *Eur J Pediatr Surg* 2010, **20**(4):242-246.

294. Ishikawa N, Kubota A, Kawahara H, Hasegawa T, Okuyama H, Uehara S, Mitani Y: **Transanal mucosectomy for endorectal pull-through in Hirschsprung's disease: comparison of abdominal, extraanal and transanal approaches**. *Pediatr Surg Int* 2008, **24**(10):1127-1129.

295. Saltzman DA, Telander MJ, Brennom WS, Telander RL: **Transanal mucosectomy: a modification of the Soave procedure for Hirschsprung's disease**. *J Pediatr Surg* 1996, **31**(9):1272-1275.

296. Vu PA, Thien HH, Hiep PN: **Transanal one-stage endorectal pull-through for Hirschsprung disease: experiences with 51 newborn patients**. *Pediatr Surg Int* 2010, **26**(6):589-592.

297. Aslanabadi S, Ghalehgolab-Behbahan A, Zarrintan S, Jamshidi M, Seyyedhejazi M: **Transanal one-stage endorectal pull-through for Hirschsprung's disease: a comparison with the staged procedures**.

298. Jia J, Yu Q, Liu G, Huang L, Li L: **[Transanal pull-through for Hirschsprung disease in new borns and infants]**. *Beijing da xue xue bao Yi xue ban = Journal of Peking University Health sciences* 2003, **35**(2):200-201.

299. Nasr A, Haricharan RN, Gamarnik J, Langer JC: **Transanal pullthrough for Hirschsprung disease: matched case-control comparison of Soave and Swenson techniques**. *J Pediatr Surg* 2014, **49**(5):774-776.

300. Jester I, Holland-Cunz S, Loff S, Hosie S, Reinshagen K, Wirth H, Ali M, Waag KL: **Transanal pull-through procedure for Hirschsprung's disease: a 5-year experience**. *Eur J Pediatr Surg* 2009, **19**(2):68-71.

301. Bing X, Sun C, Wang Z, Su Y, Sun H, Wang L, Yu X: **Transanal pullthrough Soave and Swenson techniques for pediatric patients with Hirschsprung disease**. *Medicine* 2017, **96**(10):e6209.

302. Mahajan JK, Rathod KK, Bawa M, Narasimhan KL: **Transanal Swenson's operation for recto-sigmoid Hirschsprung's disease**. *Afr J Paediatr Surg* 2011, **8**(3):301-305.

303. Levitt MA, Hamrick MC, Eradi B, Bischoff A, Hall J, Pena A: **Transanal, full-thickness, Swenson-like approach for Hirschsprung disease**. *J Pediatr Surg* 2013, **48**(11):2289-2295.

304. Ghosh DN, Liu Y, Cass DT, Soundappan SSV: **Transition zone pull-through in Hirschsprung's disease: a tertiary hospital experience**. *ANZ journal of surgery* 2017, **87**(10):780-783.

305. Witvliet MJ, Petersen N, Ekkerman E, Sleeboom C, van Heurn E, van der Steeg AFW: **Transitional health care for patients with Hirschsprung disease and anorectal malformations**. *Techniques in coloproctology* 2017, **21**(7):547-554.

306. Wei J, Zhang W, Feng JX, Weng YZ, Wei MF, Sun XY, Li N, Yu DH, Yang JX: **[Transumbilical laparoscopic pull-through for children with hypoganglionosis]**. *Zhonghua Wei Chang Wai Ke Za Zhi* 2011, **14**(10):762-763.

307. Foster P, Cowan G, Wrenn EL, Jr.: **Twenty-five years' experience with Hirschsprung's disease**. *J Pediatr Surg* 1990, **25**(5):531-534.

308. Nah SA, Ong CCP, Lie D, Marimuttu VJ, Hong J, Te-Lu Y, Low Y, Jacobsen AS: **Understanding Experiences of Youth Growing Up with Anorectal Malformation or Hirschsprung's Disease to Inform Transition Care: A Qualitative In-Depth Interview Study**. *Eur J Pediatr Surg* 2018, **28**(1):67-74.

309. Versteegh HP, Johal NS, de Blaauw I, Stanton MP: **Urological and sexual outcome in patients with Hirschsprung disease: A systematic review**. *Journal of pediatric urology* 2016, **12**(6):352-360.

310. Allin BS, Irvine A, Patni N, Knight M: **Variability of outcome reporting in Hirschsprung's Disease and gastroschisis: a systematic review**. *Scientific reports* 2016, **6**:38969.

311. Rouzrokh M, Khaleghnejad AT, Mohejerzadeh L, Heydari A, Molaei H: **What is the most common complication after one-stage transanal pull-through in infants with Hirschsprung's disease?** *Pediatr Surg Int* 2010, **26**(10):967-970.
