# Supplementary material for: Long-term outcomes and quality of life of patients with Hirschsprung disease: a systematic review and meta-analysis
Source: BMC Gastroenterol. 2020 Mar 12;20:67. doi: 10.1186/s12876-020-01208-z (PMC7066788; doi:10.1186/s12876-020-01208-z)
Supplement: Supplementary file 3 — Additional file 3. Quality appraisal of cross-sectional studies. [file 12876_2020_1208_MOESM3_ESM.docx]

**Additional file 3. Quality appraisal of cross-sectional studies**

| **Study & Year** | **Define source of information (survey, record review)** | **List inclusion and exclusion criteria for exposed and unexposed subjects (cases and controls) or refer to previous publications** | **Indicate time period used for identifying patients** | **Indicate whether or not subjects were consecutive if not population-based** | **Indicate if evaluators of subjective components of study were masked to other aspects of the status of the participants** | **Describe any assessments undertaken for quality assurance purposes (e.g., test/retest of primary outcome measurements)** | **Explain any patient exclusion from analysis** | **Describe how confounding was assessed and/or controled** | **If applicable, explain how missing data were handled in the analysis** | **Summarize patient response rates and completeness of data collection** | **Clarify what follow-up, if any, was expected and the percentage of patients for which incomplete data or follow-up was obtained** |
| --- | --- | --- | --- | --- | --- | --- | --- | --- | --- | --- | --- |
| Athanasakos et al., 2006 | Yes | Yes | Yes | Unclear | Unclear | Unclear | Yes | Unclear | Unclear | Yes | Yes |
| Mills et al., 2008 | Yes | Yes | Yes | Yes | Unclear | Unclear | Yes | Yes | No | Yes | Yes |
| Catto-Smith et al., 2007 | Yes | Yes | Yes | Yes | Unclear | Unclear | Yes | Yes | No | Yes | Yes |
| Ieiri et al., 2010 | Yes | Yes | Yes | Unclear | Unclear | Unclear | Yes | Yes | No | Yes | Yes |
| Gunnarsdo´ttir et al., 2010 | Yes | Yes | Yes | Yes | Unclear | Unclear | Yes | Yes | Yes | Yes | Yes |
| Niramis et al., 2008 | Yes | Yes | Yes | Yes | Unclear | Unclear | Yes | Yes | Unclear | No | No |
