# Supplementary material for: Long-term outcomes and quality of life of patients with Hirschsprung disease: a systematic review and meta-analysis
Source: BMC Gastroenterol. 2020 Mar 12;20:67. doi: 10.1186/s12876-020-01208-z (PMC7066788; doi:10.1186/s12876-020-01208-z)
Supplement: Supplementary file 4 — Additional file 4. [file 12876_2020_1208_MOESM4_ESM.zip › Additional file 4bR5.docx]

Additional file 4b. Egger’s graph of the publication bias of pooled prevalence of constipation
